# Photoreactive helical nanoaggregates exhibiting morphology transition on thermal reconstruction

**DOI:** 10.1038/ncomms9936

**Published:** 2015-11-20

**Authors:** Mitsuaki Yamauchi, Tomonori Ohba, Takashi Karatsu, Shiki Yagai

**Affiliations:** 1 Department of Applied Chemistry and Biotechnology, Graduate School of Engineering, Chiba University, 1-33 Yayoi-cho, Inage-ku, Chiba 263-8522, Japan; 2 Department of Chemistry, Graduate School of Science, Chiba University, 1-33 Yayoi-cho, Inage-ku, Chiba 263-8522, Japan; 3 Molecular Chirality Research Center, Chiba University, 1-33 Yayoi-cho, Inage-ku, Chiba 263-8522, Japan

## Abstract

The supramolecular design of photochromic molecules has produced various smart molecular assemblies that can switch their structures and/or functions in response to light stimuli. However, most of these assemblies require large structural changes of the photochromic molecules for an efficient conversion of assembled states, which often suppresses the photoreactivity within the self-assemblies. Here we report molecular assemblies, based on a photo-cross-linkable chromophoric dyad, in which a small amount of ultraviolet-generated photochemical product can guide the entire system into different assembly processes. In apolar solution, the intact dyad self-assembles into right-handed superhelical fibrils. On ultraviolet-irradiation of these fibrils, an effective photoreaction affords a sole photo-cross-linked product. When right-handed helical fibrils, containing a minor amount of the photoproduct, are thermally reconstructed, the intact molecule and the photoproduct undergo a co-assembly process that furnishes superhelical fibrils with different molecular packing structures. This molecular design principle should afford new paradigms for smart molecular assemblies.

## Abstract (web summary)

The ability to switch structure or function in response to an external stimulus is highly desirable for many applications. Here, the authors report the guidable supramolecular assembly of photocross-linkable molecules into different complex superstructures, dependant on their exposure to UV light.

## Graphical abstract

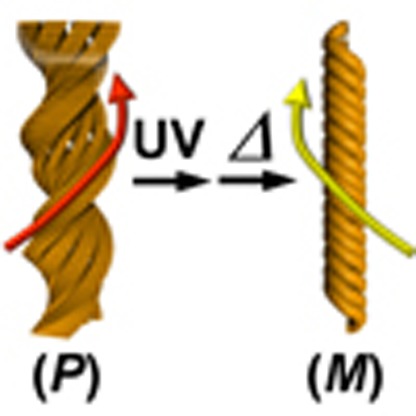

Photoreactions of naturally occurring π-conjugated molecular units can have a crucial impact on their intricate dynamic organization processes. For example, DNA bases in the duplex undergo ultraviolet-irradiation-induced photodimerization reactions between base pairs such as thymine and cytosine. Such DNA damage can, even when occurring only on a small scale, lead to severe malfunctions during the duplication and transfer of genetic information^1^. Although such photoreactions represent unfavourable events for biological systems, they enable the development of complex stimuli-responsive systems when applied in the context of synthetic supramolecular chemistry^2^.

The last decade has seen great advances in this research field, and well-defined molecular ensembles with complex hierarchy have been built^3^,^4^,^5^,^6^. However, a sophisticated molecular design that leads to a system with a well-organized thermodynamic nanostructure is not enough; for the further advancement of this research area, methodologies that enable the system to switch between different nanostructures by means of external stimuli are of equal importance^7^,^8^,^9^. A commonly encountered design strategy for the generation of stimuli-responsive molecular assemblies is the use of photochromic molecular building blocks such as azobenzenes and diarylethenes^10^,^11^,^12^. Subsequent photochromic reactions of these assemblies may affect their equilibrium, which can lead to changes in the aggregation state^13^,^14^,^15^,^16^,^17^,^18^,^19^,^20^,^21^,^22^,^23^,^24^,^25^, or to the generation of monomers^26^,^27^,^28^,^29^. To change the assembled supramolecular structures of such systems involving photochromic reactions, the majority of previously reported studies has focused on photochromic molecules with relatively large structural changes. However, photochromic reactions accompanied by large changes in molecular shape, often exhibit a suppression of photoreactivity within self-assembled nanostructures^30^,^31^. Here we report an unprecedented type of complex photoresponsive supramolecular system, wherein even minor amounts of a generated photoproduct in a self-assembled nanostructure changes the self-assembly mechanism of the intact molecules completely through the reorganization process, thus leading ultimately to distinct self-assembled nanostructures.

## Results

### Molecular design

**Figure 1:**
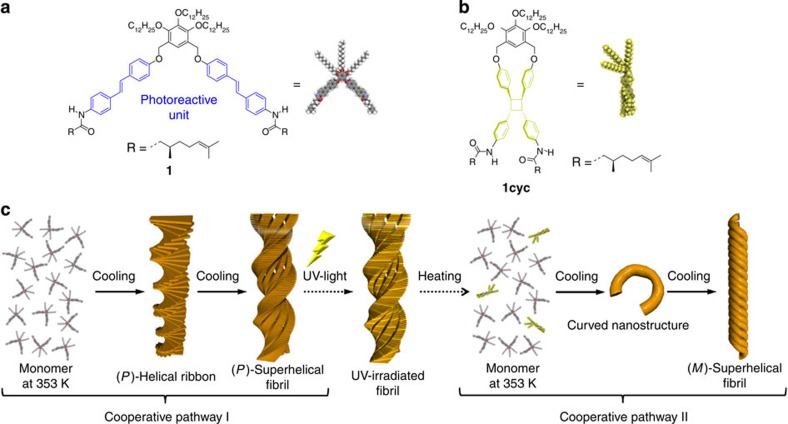
Molecular structures and assembly processes of 1 and 1cyc. (**a**,**b**) Molecular structures of the stilbene dyad **1** (**a**) and its corresponding [2+2] photocycloaddition product **1cyc** (**b**). (**c**) Proposed self-assembly process of **1** and co-assembly process of **1** and **1cyc**.

Recently, we reported a unique molecular assembly system, based on a chiral azobenzene dyad, in which the two photochromic units are linked by a xylylene^32^. This dyad exhibited a remarkable hierarchical assembly process in the nonpolar solvent methylcyclohexane (MCH). An examination of its circular dichroism (CD) behaviour suggested that intramolecular π−π stacking between two azobenzene chromophores results in a folded and thus chiral conformation of this dyad. The folded molecules further aggregated with a homogeneous curvature, which lead to the formation of uniform annular nanostructures (nanorings) at 293 K. On cooling the solution to 273 K, the annular nanostructures stacked on top of each other to form tubular nanostructures (nanotubes) that grew into chiral superhelical fibrils. In one of our previous studies, we reported that a similar dyad, consisting of two oligo(*p*-phenylenevinylenes), does not assemble into uniform nanostructures, but results in the formation of irregular nanofibrils and small amounts of nanorings^33^. We attributed the observed deterioration of the ability to form regular homogeneous aggregates to the very strong propensity of the oligo(*p*-phenylenevinylene) units to form intramolecular π−π stacks, which should decrease the conformational flexibility of the folded structure and thus hamper subsequent hierarchical organization. On the basis of these results, we postulated that the structural properties of the folded conformers are of pivotal importance to obtain well-defined nanostructures. If the structural properties are susceptible to light stimulus, then the formation of different self-assembled structures should be anticipated. Therefore, we designed and synthesized stilbene dyad **1** (Fig. 1a; for synthesis and characterization, see Supplementary Methods), in which two stilbene units can be cross-linked via a [2+2] photocycloaddition between two vinylene units in the folded conformation^34^,^35^,^36^. This photocyclization should decrease the conformational flexibility of the folded conformer (Fig. 1b), and thus induce the generation of a different self-assembled structure. We found that the intact dyad **1** can self-assemble into right-handed (*P*-type) superhelical aggregates, whereas the cross-linked dyad is unable to form well-defined nanoaggregates. Even in the presence of merely minor amounts of the photoproduct, a different co-assembly pathway emerged upon co-assembling intact and cross-linked dyads, leading to entirely different nanoaggregates, including left-handed (*M*-type) superhelical co-aggregates (Fig. 1c). This work thus can be regarded as an unprecedented example of an integrated self-sorting system induced by external stimuli^37^.

### Cooperative self-assembly of 1

**Figure 2:**
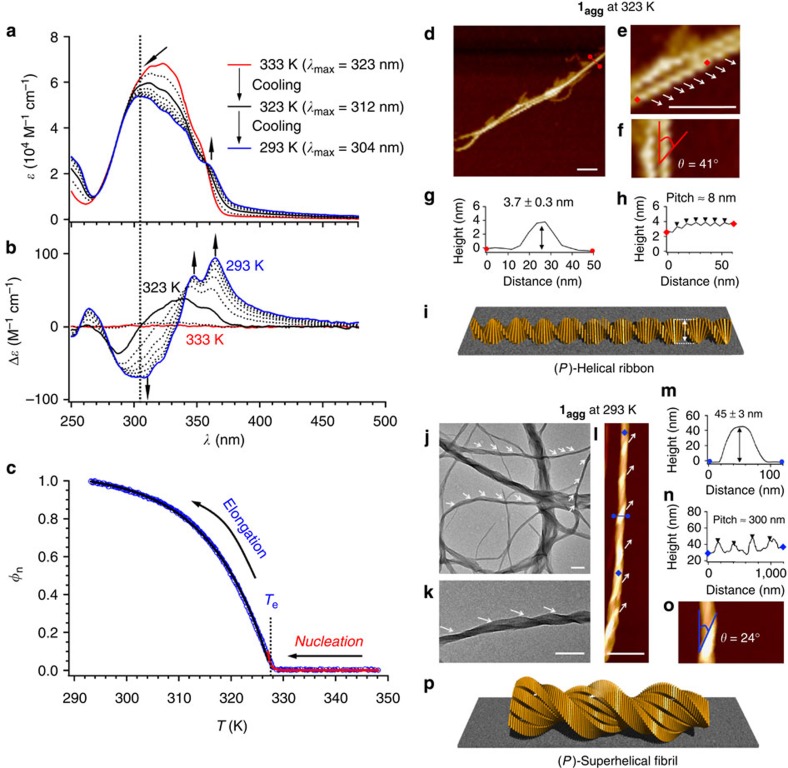
Ultraviolet (UV) absorption and CD spectroscopic, together with morphogical studies of 1. (**a**,**b**) Change of the (**a**) UV absorption and (**b**) CD spectra of **1** ([**1**]=1.0 × 10^−4^ M) in MCH upon cooling the solution from 353 to 293 K (interval: 5 K, cooling-rate: 1 K min^−1^). Since no spectral change was observed between 353 and 333 K, only the spectra for 333 K (red) and 293 K (blue) are shown for clarity. (**c**) Normalized CD intensity (*φ*_n_) at 365 nm as a function of the cooling temperature. Using a cooperative model, the red and black lines correspond to the simulated curves in the nucleation and elongation regimes, respectively. (**d**–**f**) AFM height images of (*P*)-helical ribbons of **1**_**agg**_ at 323 K. Scale bar, 50 nm. (**g**,**h**) AFM cross-sectional analysis between the red dots in images **d** and **e**. (**i**) Schematic representation of a (*P*)-superhelical ribbon. (**j**,**k**) TEM images of (*P*)-superhelical fibrils of **1**_**agg**_ found at 293 K. Scale bar, 300 nm. (**l**,**o**) AFM height images of (*P*)-superhelical fibrils of **1**_**agg**_ found at 293 K. Scale bar, 300 nm. (**m**,**n**) AFM cross-sectional analysis between the blue dots in image **l**. Samples were prepared by spin-coating MCH solutions of **1** ([**1**]=1.0 × 10^−4^ M) at appropriate temperatures onto HOPG (AFM) or carbon-coated copper grid (TEM). (**p**) Schematic representation of a (*P*)-superhelical fibril.

Chiral supramolecular aggregates of **1** were prepared by cooling hot MCH solutions of **1** ([**1**]=1.0 × 10^−4^ M) from 353 to 293 K at a cooling rate of 1 K min^−1^. At temperatures below 333 K (red curve in Fig. 2a), the absorption band at 323 nm, which is associated with the π–π* transition of *trans*-stilbene chromophores, was hypsochromically shifted to 304 nm (293 K), while an absorption shoulder emerged at *ca.* 358 nm (blue curve in Fig. 2a). Such a spectral change is typical of electronic interactions of transition dipoles between stilbene chromophores stacked in a face-to-face (H-type) arrangement^38^. In the CD spectra, a sharp growth of a bisignate CD signal with two positive maxima at 341 and 360 nm, together with a negative maximum at 287 nm (323 K) was observed initially (black curve in Fig. 2b). The zero crossing point of the bisignate signal was located at 304 nm, which is close to the absorption maximum at 323 K (dashed vertical line in Fig. 2a,b). This bisignate CD signal was indicative for a chiral exciton coupling of the stilbene chromophores with a clockwise (*P*-type) twist^39^. On further cooling the solution to 293 K, the intensity of the CD signals increased and the CD spectra became more complicated, providing two positive maxima at 348 and 365 nm and a negative maximum at 309 nm (blue curve in Fig. 2b). The observed spectral transition in the CD measurements implied a unique self-assembly process of **1** into chiral supramolecular aggregates (hereafter denoted as **1**_**agg**_).

As the self-assembly process of **1** was found to be thermally reversible, we decided to further investigate its aggregation on cooling by monitoring the increase of CD intensity at 365 nm in intervals of 0.1 K. When the increase of the normalized CD intensity (*φ*_n_), which shows the fraction of assembled molecules (*φ*_n_=1.0 for Δ*ɛ*_max_; *φ*_n_=0 for Δ*ɛ*_min_), was plotted against the temperature, a nonsigmoidal curve was obtained, which showed a sharp increase of CD activity around 328 K (Fig. 2c). Such a curve is characteristic for a nucleation–elongation (cooperative) assembly process, and the onset temperature for the elongation process (*T*_e_) was found at 327.7 K, which represents a critical temperature between (unfavourable) nucleation and (favourable) elongation^40^,^41^. This experimentally obtained curve could be fitted with a cooperative model that has been recently applied for a synthetic molecular assembly by van der Schoot, Meijer, Schenning and coworkers (Fig. 2c; for details, see Supplementary Methods)^42^. The curve fitting provided several thermodynamic parameters that characterize the assembly process of **1** (Supplementary Table 1), which include the enthalpy release in the elongation regime (Δ*H*_e_=−78.1 kJ mol^−1^) and the degree of cooperativity expressed by the activation constant (*K*_a_=2.6 × 10^−5^). The obtained negative enthalpy value shows that the cooperative assembly of **1** is an enthalpically favored process, driven by multiple noncovalent forces such as π−π stacking and hydrogen-bonding. The low *K*_a_ value (*K*_a_<<1) is indicative of a high degree of cooperativity in the formation process of **1**_**agg**_. It is worth mentioning that the cooperative assembly of **1** was maintained on decreasing the concentration to 0.4 × 10^−4^ M, while *T*_e_ was decreased to 318 K (Supplementary Fig. 1, Supplementary Table 1).

### (*P*)-helical aggregates of 1

Atomic force microscopy (AFM) and transmission electron microscopy (TEM) observations revealed that the cooperative assembly of **1** leads to well-defined superhelical nanostructures with one-handed helicity **(1**_**agg**_). To visualize nanostructures formed in different regimes of the cooperative assembly model described above, solutions of **1** ([**1**]=1.0 × 10^−4^ M) were spin coated at either 323 K (just below *T*_e_) or 293 K (<<*T*_e_) onto highly oriented pyrolytic graphite (HOPG). Dynamic light scattering (DLS) measurements of the solution at 323 K revealed the presence of aggregates with an average hydrodynamic diameter (*D*_H_) of *ca*. 250 nm (Supplementary Fig. 2). The AFM images of the sample prepared from the solution at 323 K showed the formation of well-defined ribbon-like nanostructures with exclusively right-handed helicity, that is, (*P*)-helical ribbons (Fig. 2d). The extended (*P*)-helical morphology is consistent with the local helical stacking deduced from the CD measurements that showed a *P*-type twisting of the stilbene chromophore on stacking (see black curve in Fig. 2b). These (*P*)-helical ribbons have an average thickness of 3.7±0.3 nm (Fig. 2g), which is in good agreement with the molecular length of folded **1** (*ca*. 4 nm; see Supplementary Fig. 3). The angle (*θ*) and helical pitch of the ribbons are ∼41° and 8 nm, respectively (Fig. 2e,f,h). On the basis of the thickness of a molecular model for a folded conformer of **1** (Supplementary Fig. 3) and assuming a π−π stacking distance of 0.35 nm, the helical structure should consist of ∼11 stacked molecules of folded **1** per pitch (Fig. 2i).

Further cooling of the solution to 293 K resulted in the formation of very large aggregates, whose *D*_H_ values could not be extracted by DLS, and the formation of higher order helical fibrils with an average thickness of 45±3 nm was confirmed by TEM and AFM imaging (Fig. 2j–o). These higher order fibrils also exhibited right-handed superhelicity ((*P*)-superhelical fibrils, Fig. 2p) with helical pitches of ∼300 nm (Fig. 2n) and helical angles (*θ*) of *ca*. 24° (Fig. 2o). Although the AFM analysis showed the formation of two distinct helical nanostructures, the CD cooling curve in the elongation regime is composed of a single elongation process (see Fig. 2c). This result was corroborated by a temperature-dependent ultraviolet absorption study, which also displayed a typical cooperative curve based on a single elongation regime (Supplementary Fig. 4). On the basis of these results, we concluded that the superhelical fibrils should only be formed from the helical ribbons at the very end of the elongation regime, and consequently their formation should not be reflected in the cooling curves.

### Photoreactions within helical aggregates

Given that **1** adopts a folded conformation (Supplementary Fig. 3) in the supramolecular self-aggregates, the configuration of the stilbene chromophores should be suitable for intramolecular [2+2] photocycloadditions^43^,^44^,^45^,^46^. The molecular model for folded **1** showed that the distance between the two vinylene groups of two adjacent stilbene moieties was ∼0.35 nm, which is an appropriate distance for [2+2] photocycloadditions (Supplementary Fig. 3).

**Figure 3:**
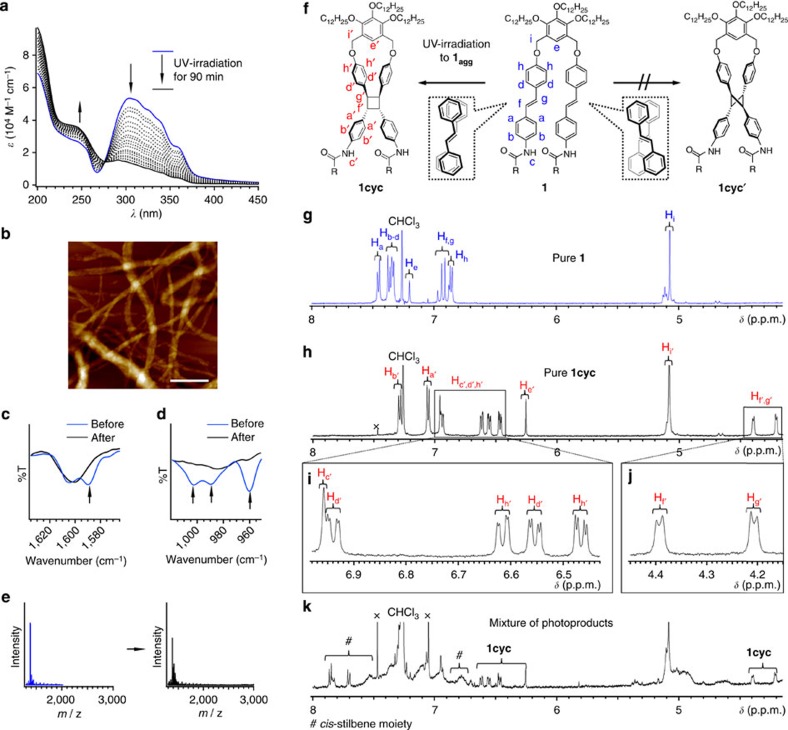
Analysis of the photoproducts formed by exposing 1_agg_ to ultraviolet (UV)-irradiation. (**a**) UV absorption spectra of a MCH solution of **1** ([**1**]=1.0 × 10^−4^ M) at 293 K upon UV-irradiation (365 nm, 90 min). The photoconversion yield for **1cyc** (80%) was determined by ^1^H NMR analysis. (**b**) AFM height image of aggregates (**1**:**1cyc**=20:80) spin-coated from a MCH solution onto HOPG. Scale bar, 500 nm. (**c**,**d**) FT/IR spectra of **1**_**agg**_ (blue) and aggregates (**1**:**1cyc**=20:80; black). Film samples for infreared spectroscopy were prepared by drop-casting MCH solutions (before and after UV-irradiation for 90 min) onto substrates. (**e**) ESI-MS spectra of **1** (blue) and 20:80 mixture of **1** and **1cyc** (black). (**f**) Possible intramolecular photo-cross-linking reactions of **1** with assignment of the corresponding ^1^H NMR signals. (**g**) ^1^H NMR spectrum of **1** in CDCl_3_. (**h**–**j**) ^1^H NMR spectra of **1cyc** in CDCl_3_ after UV-irradiation in MCH. Pure **1cyc** was prepared by UV-irradiation of a MCH solution ([**1**]=1.0 × 10^−3^ M) at 333 K for 1 h. (**k**) ^1^H NMR spectrum of a mixture of photoproducts in CDCl_3_ after UV-irradiation in CHCl_3_.

When helical fibrils of **1**, dispersed in MCH, were exposed to irradiation with ultraviolet light (365 nm), the absorption band associated with the *trans*-stilbene chromophore decreased, concomitant with the emergence of broad absorption bands below 255 nm (Fig. 3a)^47^. The spectral change of the absorption leveled off after irradiation for 90 min, and the ^1^H nuclear magnetic resonance (NMR) analysis suggested the formation of a single photoproduct in 80% yield (Supplementary Fig. 5). The CD measurements showed a decrease of the bisignate signals as a result of the irradiation-induced bleaching of the stilbene chromophores (Supplementary Fig. 6). On spin coating this ultraviolet-irradiated solution onto HOPG, we found fibrillar nanostructures in quantities that were almost identical to those of **1**_**agg**_ (Fig. 3b, Supplementary Fig. 7). However, the resulting fibres exhibited ambiguous surfaces, which were clearly different from the well-defined superhelical structures of **1**_**agg**_. On the basis of these observations, we propose that the superhelical fibrils are microscopically phase-separated during their formation, and that the majority of photoproduct (hereafter denoted as **1cyc**) formed on ultraviolet-irradiation is confined within the superhelical structures. Only a minor amount of **1cyc**, probably located on the fiber surface, might be partially dissolved.

Fourier-transform infrared (FT-IR) spectra of the cast films prepared from the solutions before **(1**_**agg**_) and after exposure to 90 min of ultraviolet-irradiation exhibited a decrease of the C=C stretching band at 1,590 cm^−1^ and the C=C−H out-of-plane bending bands at 1,002, 989, and 960 cm^−1^, which are all associated with the vinylene group of the stilbene moieties (Fig. 3c,d)^48^. The possibility of intermolecular photoreactions of **1** can be excluded, based on the fact that only monomeric species (*m*/*z*=1,382) were detected in electrospray ionization mass (ESI-MS) measurements of the ultraviolet-irradiated solution. Conversely, dimeric or oligomeric species could not be detected (Fig. 3e). These results corroborate the occurrence of an intramolecular photoreaction, which in turn indirectly confirms an assembly mechanism of **1** through intramolecular folding. The absence of intermolecular [2+2] photocycloadditions suggests that the intermolecular stacking arrangement of adjacent folded **1** is not suitable for such photoreactions. This is most likely due to a rotational offset and consistent with the helical morphology of **1**_**agg**_ (Fig. 2d−f).

The ^1^H NMR analysis of the photoproduct in CDCl_3_ provided not only evidence for the formation of an intramolecular [2+2] cycloadduct **(1cyc**) but also insight into its steric configuration. As we were unable to characterize the photoproduct by ^1^H NMR from the ultraviolet-irradiated sample (90 min) used for the above analyses (Supplementary Fig. 5), we attempted to obtain a pure sample of the photoproduct via modified ultraviolet-irradiation conditions. A concentrated **1**_**agg**_ solution ([**1**]=1.0 × 10^−3^ M) that was exposed to ultraviolet-irradiation while being gently heated to 333 K provided the photoproduct in almost quantitative yield. The ^1^H NMR spectrum of this material displayed a single set of signals, in which all resonances in the aromatic region were subject to high-field shifts relative to those of intact **1** (Fig. 3g−j). The two doublets observed in the aliphatic region (H_f_′ and H_g_′) were ascribed to a cyclobutane ring^49^,^50^. The chemical shifts and the coupling constants of these doublets (*δ*=4.20 and 4.39 p.p.m.; *J*=5.9 Hz) suggest that **1cyc** contains a stereoselectively formed cyclobutane ring with two pairs of *cis*-configurated phenyl groups. On the basis of these results, the presence of a photoisomer, in which the cyclobutane ring possesses four phenyl groups in all-*trans* configuration **(1cyc′**), can thus be excluded (Fig. 3f). The stereoselective photoreaction demonstrated that **1** adopts a specific chiral conformation in the fibrillar aggregates (Fig. 3f). Evidence supporting this notion was obtained in form of a complex ^1^H NMR spectrum, reflecting the formation of several photoproducts including isomerized products as well as **1cyc**, when ultraviolet-irradiation experiments for monomeric **1** were carried out in CDCl_3_ (Fig. 3k).

### Reconstruction of ultraviolet-exposed aggregates

**Figure 4:**
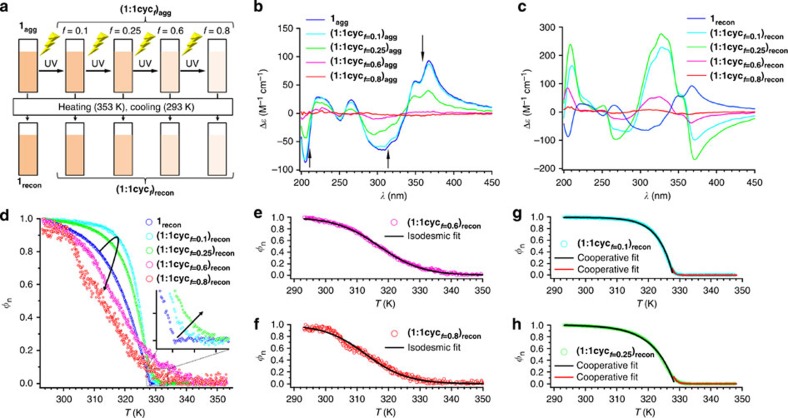
CD studies for the reconstruction processes of co-aggregates consisting of 1 and 1cyc. (**a**) Schematic representation of the conversion procedure from **1**_**agg**_ to **(1:1cyc**_***f***_**)**_**recon**_. (**b**,**c**) CD spectra of mixtures of **1** and **1cyc** in MCH (total concentration=1.0 × 10^−4^ M) at 293 K (**b**) before [**(1:1cyc**_***f***_**)**_**agg**_] and (**c**) after thermal annealing [**(1:1cyc**_***f***_**)**_**recon**_]. (**d**) Normalized CD intensity at 370 nm (*φ*_n_) as a function of the temperature in the reconstruction processes of **(1:1cyc**_***f***_**)**_**recon**_ from 353 to 293 K (cooling rate: 1 K min^−1^). The arched arrow indicates the transition of the cooling-curves of **1** on increasing the fraction of **1cyc**. The inset shows magnified plots in the nucleation regimes close to *T*_e_, and the arrow indicates the transition of the cooling curves of **1** on increasing the fraction of **1cyc**. (**e**,**f**) Isodesmic model fitting of the cooling curves of **(1:1cyc**_***f*****=0.6**_**)**_**recon**_ and **(1:1cyc**_***f*****=0.8**_**)**_**recon**_. (**g**,**h**) Cooperative model fitting of the cooling curves of **(1:1cyc**_***f*****=0.1**_**)**_**recon**_ and **(1:1cyc**_***f*****=0.25**_**)**_**recon**_.

We observed that photoproduct **1cyc** is able to markedly influence the self-assembly process of **1** through thermal reconstruction, giving rise to the evolution of helical nanofibers with a chiral sense that is diametrically opposed to that of **1**_**agg**_. To determine the effect of the content of **1cyc** on the self-assembly of **1**, we varied the yield of the photocycloaddition product by modulating the ultraviolet-irradiation time for **1**_**agg**_ ([**1**]=1.0 × 10^−4^ M; Fig. 4a and Supplementary Fig. 8). After ultraviolet-irradiation, solutions were heated to 353 K to dissociate all aggregates, before they were cooled to 293 K at a cooling rate of 1 K min^−1^. Accordingly, we obtained **1**_**agg**_, containing different molar fractions of **1cyc** (hereafter denoted as **(1:1cyc**_***f*****=0.1–0.8**_**)**_**agg**_, wherein the subscript *f* indicates the molar fraction of **1cyc**), and thermally reconstructed co-aggregates of **1** and **1cyc** (denoted as **(1:1cyc**_***f*****=0.1–0.8**_**)**_**recon**_**)**, containing different molar fractions of **1cyc** (*f*=0.1, 0.25, 0.6, and 0.8).

Figure 4b,c compare CD spectra of **(1:1cyc**_***f*****=0.1–0.8**_**)**_**agg**_ and **(1:1cyc**_***f*****=0.1–0.8**_**)**_**recon**_. The reconstructed co-aggregates with low proportions of **1cyc**, that is, **(1:1cyc**_***f*****=0.1**_**)**_**recon**_ and **(1:1cyc**_***f*****=0.25**_**)**_**recon**_, displayed CD signals with large positive peaks at 209 and 327 nm, as well as negative peaks at 268 and 372 nm (Fig. 4c), which are considerably different from the CD signals of the corresponding **(1:1cyc**_***f***_**)**_**agg**_ (Fig. 4b). The positive and negative signs in the longer wavelength region indicated a counterclockwise helicity (*M*-type) for the stilbene chromophores. The CD signals of **(1:1cyc**_***f*****=0.1**_**)**_**recon**_ and **(1:1cyc**_***f*****=0.25**_**)**_**recon**_ are not mirror images of those of **1**_**agg**_, and the CD intensities of the reconstructed co-aggregates were notably higher than those of the self-aggregates (Fig. 4c). For example, the anisotropy factor (|*g*|=Δ*ɛ*/*ɛ*) of **1** in **1**_**agg**_ was 0.0046 (at 364 nm), which increased to 0.012 (at 372 nm) in **(1:1cyc**_***f*****=0.1**_**)**_**recon**_. This remarkable **1cyc**-induced CD enhancement suggested that the self-assembly of **1** could be largely affected by **1cyc**. Further increasing the **1cyc** fraction to 0.6 and 0.8 [**(1:1cyc**_***f*****=0.6**_**)**_**recon**_ and **(1:1cyc**_***f*****=0.8**_**)**_**recon**_] weakened the CD intensity (Fig. 4c), which demonstrated that the new CD signals do not originate from the self-assembly of **1cyc**.

To gain further insight into how the self-assembly process of **1** can be affected by **1cyc**, we monitored the increase of the CD activity at 370 nm on the reconstruction process, that is, during cooling (Fig. 4d). The **1cyc**-rich reconstructed co-aggregates **(1:1cyc**_***f*****=0.6**_**)**_**recon**_ and **(1:1cyc**_***f*****=0.8**_**)**_**recon**_ displayed sigmoidal cooling curves. These curves could be fitted with an isodesmic (equal *K*) model, wherein the whole assembly process is governed by a single equilibrium constant (*K*_iso_; Fig. 4e,f; for details, see Supplementary Methods)^51^. The theoretical fitting afforded thermodynamic parameters including enthalpy release (Δ*H*_iso_), *K*_iso_ in the elongation regime, and the melting temperature *T*_m_ at which *φ*_n_=0.5 (Supplementary Table 2). The *K*_iso_ values of **(1:1cyc**_***f*****=0.6**_**)**_**recon**_ and **(1:1cyc**_***f*****=0.8**_**)**_**recon**_ at 298 K are 8.6 × 10^4^ and 8.3 × 10^4^ M^−1^, respectively. It is noteworthy that, as described previously, the cooperative assembly of pure **1** (to give **1**_**agg**_) was maintained on decreasing its concentration to the same concentration of **1** as in **(1:1cyc**_***f*****=0.6**_**)**_**recon**_ (0.4 × 10^−4^ M), which revealed the occurrence of co-aggregation through the reconstruction process.

Conversely, for the reconstructed co-aggregates with low proportions of **1cyc**, that is, **(1:1cyc**_***f*****=0.1**_**)**_**recon**_ and **(1:1cyc**_***f*****=0.25**_**)**_**recon**_, cooperative curves were obtained in the cooling process (Fig. 4g,h). However, these curves are different from those of **1**_**agg**_, and reflect a more moderate nucleation process (inset in Fig. 4d), followed by a steeper progression of the elongation process (Fig. 4d). These findings suggested that these co-aggregates are formed through processes with lower degrees of cooperativity, but with larger enthalpy changes in the elongation regime. Indeed, a theoretical fitting of these curves to the nucleation–elongation model provided thermodynamic parameters *T*_e_, Δ*H*_e_, and *K*_a_ (Supplementary Table 3). The value of *K*_a_, which reflects degree of cooperativity, was found to increase from 2.6 × 10^−5^ for **1**_**agg**_ to 5.9 × 10^−4^ for **(1:1cyc**_***f*****=0.1**_**)**_**recon**_. Furthermore, co-aggregates **(1:1cyc**_***f*****=0.1**_**)**_**recon**_ exhibited a more negative enthalpy release (Δ*H*_e_=−187.8 kJ mol^−1^) relative to that of **1**_**agg**_ (Δ*H*_e_=−78.1 kJ mol^−1^), thus revealing a stronger binding of the nuclei in the elongation regime of the co-aggregates, despite of the decrease of the stilbene chromophore concentration in the entire system. Accordingly, our analysis demonstrated that even a small amount of photoproduct **1cyc** enabled **1** to assemble into different co-aggregates^52^,^53^,^54^.

### Nanostructures of reconstructed co-aggregates

**Figure 5:**
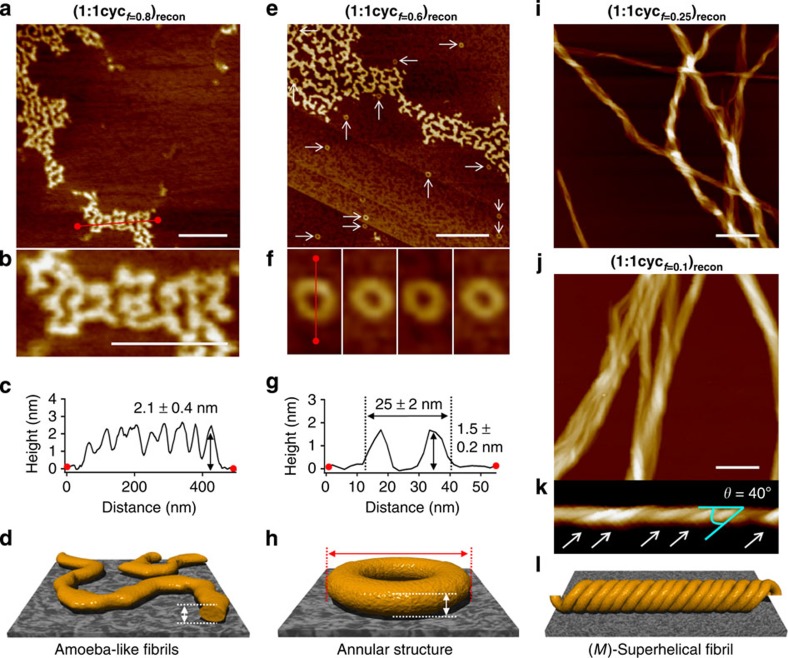
Morphologies of co-aggregates consisting of 1 and 1cyc. (**a**,**b**) AFM height images, (**c**) AFM cross-sectional analysis between the red dots in image **a**, (**d**) cartoon representation of the observed nanostructure of **(1:1cyc**_***f*****=0.8**_**)**_**recon**_ spin-coated from a MCH solution (total concentration=1.0 × 10^−4^ M) onto HOPG. Scale bar, 300 nm. (**e**,**f**) AFM height images, (**g**) AFM cross-sectional analysis between the red dots in image **f**, (**h**) cartoon representation of the observed nanostructure of **(1:1cyc**_***f*****=0.6**_**)**_**recon**_ spin-coated from a MCH solution (total concentration=1.0 × 10^−4^ M) onto HOPG. Scale bar, 300 nm. (**i**) AFM height images of the observed nanostructure of **(1:1cyc**_***f*****=0.25**_**)**_**recon**_ spin-coated from a MCH solution (total concentration=1.0 × 10^−4^ M) onto HOPG. Scale bar, 300 nm. (**j**,**k**) AFM height images, and (**l**) cartoon representation of the observed nanostructure of **(1:1cyc**_***f*****=0.1**_**)**_**recon**_ spin-coated from a MCH solution (total concentration=1.0 × 10^−4^ M) onto HOPG. Scale bar, 300 nm.

As the CD studies revealed reconstruction processes with different cooperativity depending on the **1cyc** content (Fig. 4), we analysed the resulting nanostructures by AFM. Initially, we studied nanostructures formed by pure **1cyc** prepared under special conditions (see Fig. 3), and confirmed that **1cyc** is able to form only amorphous small aggregates (*D*_H_=*ca*. 50 nm in MCH) without CD activity (Supplementary Fig. 9). AFM images of co-aggregates **(1:1cyc**_***f*****=0.8**_**)**_**recon**_, obtained from solutions with a combined concentration of 1.0 × 10^−4^ M and reconstructed by the isodesmic mechanism, displayed irregular amoeba-like structures with an average thickness and width of 2.1±0.4 and ∼20 nm, respectively (Fig. 5a–d). The DLS analysis of the reconstructed solution revealed the presence of particles with an average *D*_H_ of *ca*. 400 nm, and thus suggested that these ill-defined aggregates were already formed in solution (Supplementary Fig. 10). It is worth noting that helical fibrillar nanostructures were not encountered for these co-aggregates, although pure **1** afforded elongated nanofibrils even at a concentration of 2.0 × 10^−5^ M (Supplementary Fig. 11). These findings demonstrated that minor amounts of **1** are incorporated into the amoeba-like nanostructures, and that self-sorting through the reconstruction process does not occur between **1** and **1cyc**^55^. The formation of ill-defined morphologies by this **1cyc**-rich co-aggregated system showed that the photoreaction of **1** deteriorates its aggregation ability, most likely due to decreased conformational flexibility of the folded structure. A similar observation was made for the previously reported oligo(*p*-phenylenevinylene) dyad^33^, which produced only irregular fibrils, due to the decreased conformational flexibility of the folded conformer by strong intramolecular π−π stacking.

On decreasing the molar fraction of **1cyc** to 0.6 [**(1:1cyc**_***f*****=0.6**_**)**_**recon**_], a small amount of annular nanostructures emerged in addition to the amoeba-like fibrils (Fig. 5e–h). DLS measurements confirmed the simultaneous formation of annular (*D*_H_=*ca*. 30 nm) and fibrillar aggregates (*D*_H_=*ca*. 450 nm; Supplementary Fig. 10). For these annular nanostructures, an average outside diameter and thickness of 25±2 and 1.5±0.2 nm were observed, respectively. Similar well-defined and closed nanostructures were not encountered for the aggregation of individual components of either **1** (Fig. 2) or **1cyc** (Supplementary Fig. 9a,b). Even more interestingly, a further decrease of the molar fraction of **1cyc** to 0.25 [**(1:1cyc**_***f*****=0.25**_**)**_**recon**_] and 0.1 [**(1:1cyc**_***f*****=0.1**_**)**_**recon**_] resulted in the generation of left-handed (*M*-type) superhelical fibrils (Fig. 5i–l, Supplementary Fig. 12) with a helical angle (*θ*) of 40° (Fig. 5k). When considering these results in combination with those of the CD studies (Fig. 4b,c), it becomes clear that **1cyc** alters the self-assembly mechanism of **1** drastically, affording superhelical structures with a diametrically opposed chirality to that of **1**_**agg**_.

**Figure 6:**
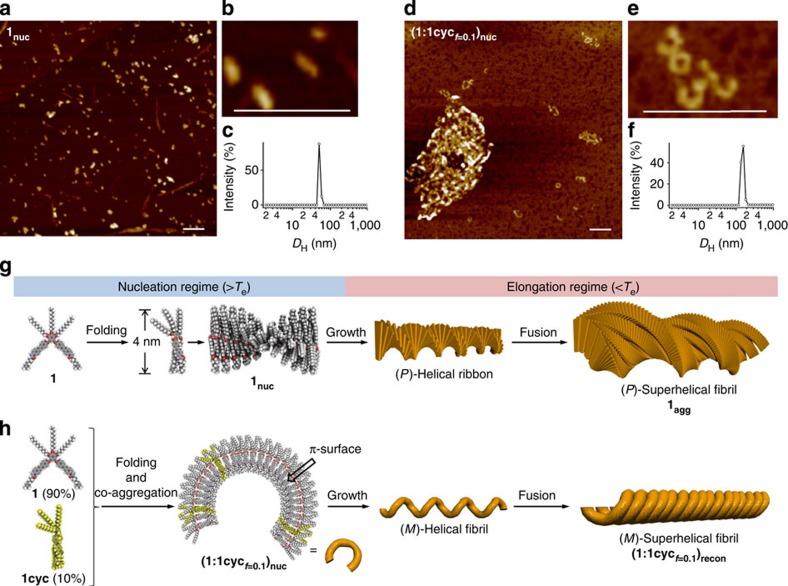
Comparison of the cooperative assembly processes of 1_agg_ and (1:1cyc_*f*=0.1_)_recon_. (**a**,**d**) AFM height images of **1**_**nuc**_ and **(1:1cyc**_***f*****=0.1**_**)**_**nuc**_, respectively. Scale bar, 150 nm. (**b**,**e**) Magnified AFM images of typical nanostructures in (**a**,**d**), respectively. Scale bar, 150 nm. Samples were prepared by spin-coating MCH solutions (total concentration=1.0 × 10^−4^ M) at 328 K (*T*_e_) onto HOPG. (**c**,**f**) DLS-derived size distribution of aggregates of **1**_**nuc**_ and **(1:1cyc**_***f*****=0.1**_**)**_**nuc**_ in MCH, respectively. (**g**,**h**) Schematic representation of the proposed cooperative assembly processes of **1**_**agg**_ and **(1:1cyc**_***f*****=0.1**_**)**_**recon**_, respectively.

To further investigate the observed difference between the two cooperative assembly pathways of **1**_**agg**_ and **(1:1cyc**_***f*****=0.1**_**)**_**recon**_, we studied the morphologies of the ‘nuclei' **1**_**nuc**_ and **(1:1cyc**_***f*****=0.1**_**)**_**nuc**_, which we suspected to be responsible for the formation of these (co)aggregates. For this purpose, AFM measurements were carried out on samples obtained from spin-coating aggregate solutions heated to *T*_e_ (328 K) onto HOPG. AFM images of **1**_**nuc**_ displayed a number of non-uniform particles, together with a small amount of extended helical fibrils (Fig. 6a,b). In strong contrast, AFM analysis of **(1:1cyc**_***f*****=0.1**_**)**_**nuc**_ revealed agglomerates of curved nanostructures including annular structures (Fig. 6d,e). This result was supported by DLS measurements that showed different aggregate sizes for **1**_**nuc**_ and **(1:1cyc**_***f*****=0.1**_**)**_**nuc**_ (Fig. 6c,f). The result of **(1:1cyc**_***f*****=0.1**_**)**_**nuc**_ demonstrated that the formation of curved nanostructures should be involved in the nucleation process of the (*M*)-superhelical fibrils. Accordingly, we would like to propose the cooperative assembly mechanisms shown in Fig. 6g,h for **1**_**agg**_ and **(1:1cyc**_***f*****=0.1**_**)**_**recon**_, respectively.

In the absence of **1cyc**, **1** self-assembles via intramolecular folding to form helical nuclei **(1**_**nuc**_, Fig. 6g). The bisignate CD signal of **1** observed at 323 K (see black curve in Fig. 2b), which is slightly below *T*_e_ (328 K), can be attributed to oligomeric (*P*)-helical stacks composed of chirally folded **1** that also adopts a (*P*)-helical conformation. In the elongation regime below *T*_e_, these helical **1**_**nuc**_ nuclei grow exponentially into (*P*)-helical ribbons, which further aggregate to form higher order (*P*)-superhelical fibrils, leading to an enhanced CD intensity (Fig. 6g). In the presence of minor amounts of **1cyc**, **1** is assumed to pursue the same folding–assembling process. However, extended helical aggregation should be compromised by covalently folded **1cyc**, which is unable to form well-defined aggregates, but can induce strong curvature on co-aggregation. In such **1cyc**-doped systems, curved nuclei **(1:1cyc**_***f*****=0.1**_**)**_**nuc**_ should accordingly be formed (Fig. 6h). This co-aggregation motif is similar to the aggregation of our previously reported azobenzene dyad forming annular nanostructures^32^. Due to the non-helical stacking of the co-aggregates, the resulting nuclei must have π−π interactive surfaces, and can thus aggregate further on extension through π−π interaction (Fig. 6h). FT-IR measurements indicated that hydrogen bonding between amide groups facilitates the formation of both (*P*)- and (*M*)-superhelical aggregates (Supplementary Fig. 13). Due to the unique organization process of **(1:1cyc**_***f*****=0.1–0.25**_**)**_**recon**_, which is completely different from that of **1**_**agg**_, the molecular chirality of **1** is transferred to the higher order structures via a different mechanism, giving rise to the formation of (*M*)-superhelical fibrils, which exhibit complicated CD spectra as a result of multiple exciton coupling.

## Discussion

The spectroscopic and microscopic studies described so far unveiled complex self-assembly pathways for **1**, which can be affected by co-assembling with its photoproduct **1cyc**. In Supplementary Fig. 14, three possible self- and co-assembling pathways for **1** and **1cyc** are outlined, whereby the energy diagrams are based on the spectroscopic studies. Self-aggregation of **1** via intramolecular folding and extended helical stacking (Supplementary Fig. 14a) is significantly different from the annular supramolecular polymerization of the azobenzene analogue^32^. The extended helical aggregation of **1** might be caused by a stronger π−π stacking ability of the stilbene chromophore compared with the azobenzene chromophore. Based on the observed *K*_a_ and *T*_e_ values for **1**, we obtained a free energy change of nucleation (Δ*G*_n_) of 3.7 kJ mol^−1^ at *T*_e_ (Supplementary Fig. 14b)^56^. The positive Δ*G*_n_ value indicated that this cooperative pathway involves an unfavourable nucleation process, in which uphill nucleation and downhill elongation processes can be clearly distinguished. In sharp contrast, pure **1cyc** only provided amorphous aggregates, due to impaired π−π stacking ability and conformational flexibility. However, when co-assembling with varying minor amounts of **1**, **1cyc** can form either amoeba-like fibrils or annular nanostructures (Supplementary Fig. 14c) via non-cooperative (isodesmic) assembly processes (Supplementary Fig. 14d). The lack of cooperativity in these **1cyc**-rich systems can be rationally explained on the basis of a dilution effect of **1** by **1cyc**, which prevents **1** from nucleating. When **1cyc** is the minor component, the mixtures co-assembled in parallel using another cooperative pathway, in which the cooperativity is lower than that of **1**_**agg**_ (Supplementary Fig. 14e). For the **1cyc**-poor co-aggregates, Δ*G*_n_ values of −4.8 and −5.6 kJ mol^−1^ for **(1:1cyc**_***f*****=0.1**_**)**_**recon**_ and **(1:1cyc**_***f*****=0.25**_**)**_**recon**_, respectively, were calculated from *K*_a_ and *T*_e_ values (Supplementary Fig. 14f). These negative Δ*G*_n_ values uniquely characterize the assembly process of the co-aggregates as a cooperative downhill assembly model with ‘favourable' nucleation^41^. The generation of energetically more stable nuclei is probably due to a stronger interchain association of helically folded quasi-one-dimensional supramolecular polymer chains (Fig. 6h).

Ultraviolet-irradiation of the reconstructed aggregates at 250 nm did not regenerate the stilbene moieties via the retrophotoreaction and we could accordingly not obtain the original supramolecular assemblies. Our present system is nevertheless highly remarkable, as multicomponent self-assembled systems are often subject to narcissistic self-sorting, resulting in the formation of thermodynamic product mixtures. Although integrative social self-sorting systems are abundant in natural molecular assemblies, the occurrence of such sophisticated molecular ensembles in synthetic systems strongly relies on specific noncovalent interactions. The successful integration of intact molecules and photoproducts into the same self-assembled nanosystem through reconstruction might be due to small alterations of the molecular shape before and after the photoreaction^37^. A similar approach could be applied to other photoreactive units with proper covalent linkages. Our results thus provide new insights into multicomponent self-assembled systems, the morphologies and properties of which could be tuned by modulating the mixing ratio of the two constituent components.

## Methods

### Materials

All starting materials and reagents were purchased from commercial suppliers. Compound **1** was synthesized according to synthetic scheme in Supplementary Methods. Characterization of **1** and its photoproduct **1cyc** was achieved by ^1^H (Supplementary Figs 15 and 18), ^13^C (Supplementary Fig. 16), and 2D-COSY (Supplementary Figs 17 and 19) NMR spectroscopy, in combination with ESI-MS.

### Atomic force microscopy

AFM images were acquired under ambient conditions using a Multimode 8 Nanoscope V microscope (Bruker Instruments) in peak force tapping (Scanasyst) mode. Silicon cantilevers (SCANASYST-AIR) with a spring constant of 0.4 N m^−1^ and frequency of 70 kHz (nominal value, Bruker, Japan) were used in peak force tapping mode. Samples were prepared by spin-coating assembly solutions onto freshly cleaved HOPG.

### Photoirradiation experiments

Photoirradiation experiments were performed using 365-nm ultraviolet light from a LED lamp (17 mW cm^−2^). Sample solutions in a quartz cuvette were placed at the distance of 5 cm from the light source.

## Additional information

**How to cite this article:** Yamauchi, M. *et al*. Photoreactive helical nanoaggregates exhibiting morphology transition on thermal reconstruction. *Nat. Commun.* 6:8936 doi: 10.1038/ncomms9936 (2015).

## Supplementary Material

Supplementary Information Supplementary Figures 1-19, Supplementary Table 1-3, Supplementary Methods and Supplementary References
