# Supplementary material for: Photoreactive helical nanoaggregates exhibiting morphology transition on thermal reconstruction
Source: Nat Commun. 2015 Nov 20;6:8936. doi: 10.1038/ncomms9936 (PMC4673833; doi:10.1038/ncomms9936)
Supplement: Supplementary Information — Supplementary Figures 1-19, Supplementary Table 1-3, Supplementary Methods and Supplementary References [file ncomms9936-s1.pdf]

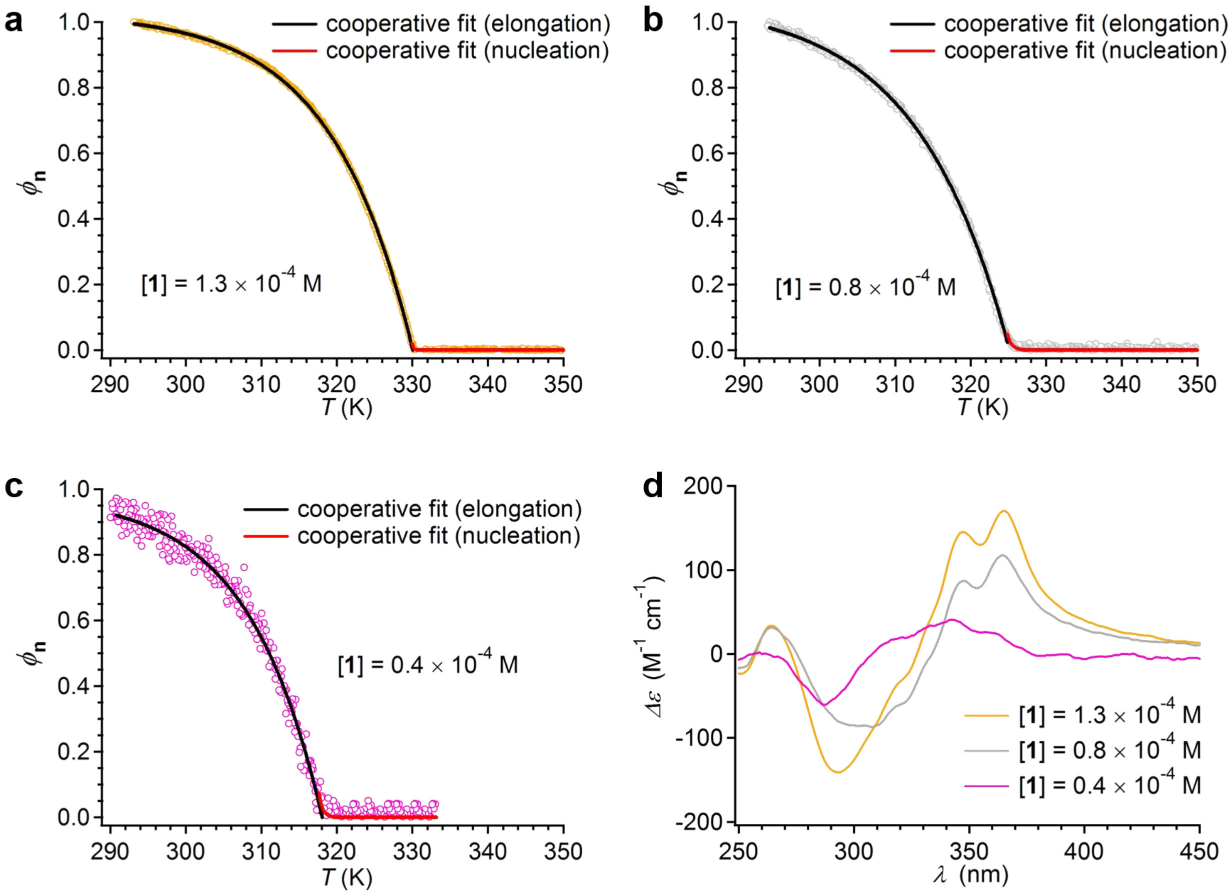

Supplementary Figure 1. Concentration-dependent cooling curves and CD spectra of 1. (**a**–**c**) Normalized CD intensity (ϕ_n) at 365 nm as a function of the temperature, obtained by monitoring the CD intensity of MCH solutions of **1** at three different concentrations upon cooling hot monomeric solutions to 293 K with a cooling rate of 1 K·min^{-1}. Solid curves represent theoretical fitting traces based on a cooperative model (assembly model). (**d**) CD spectra of MCH solutions of **1** at three different concentrations (293 K).

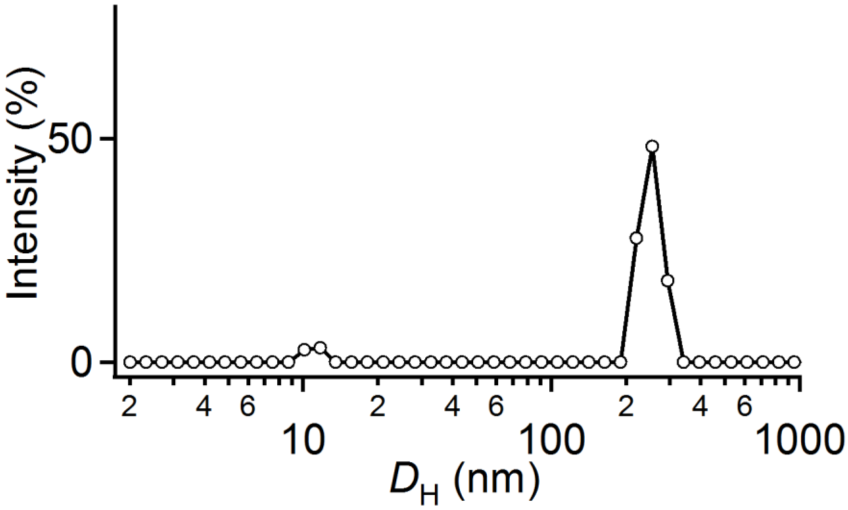

Supplementary Figure 2. DLS result for (*P*)-helical ribbons of 1 at 323 K. DLS result of (*P*)-helical ribbons of **1** in MCH at 323 K.

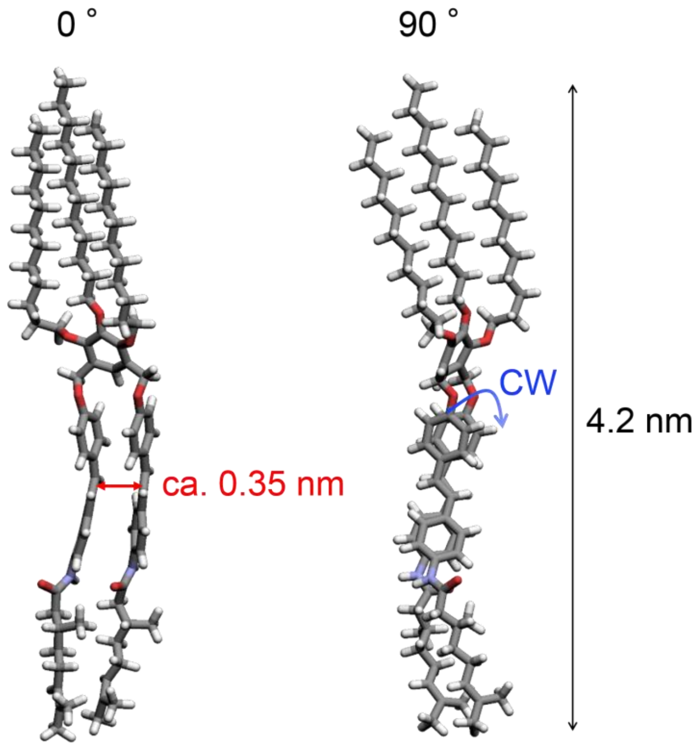

Supplementary Figure 3. Proposed foldamer geometry of 1. Optimized geometry for the folded conformation of **1** with minimized energy, in which two stilbene chromophores are stacked in a clockwise (*P*-type) rotational displacement. A typical distance of *ca*. 0.35 nm between the C=C bonds of stilbene moieties was observed.

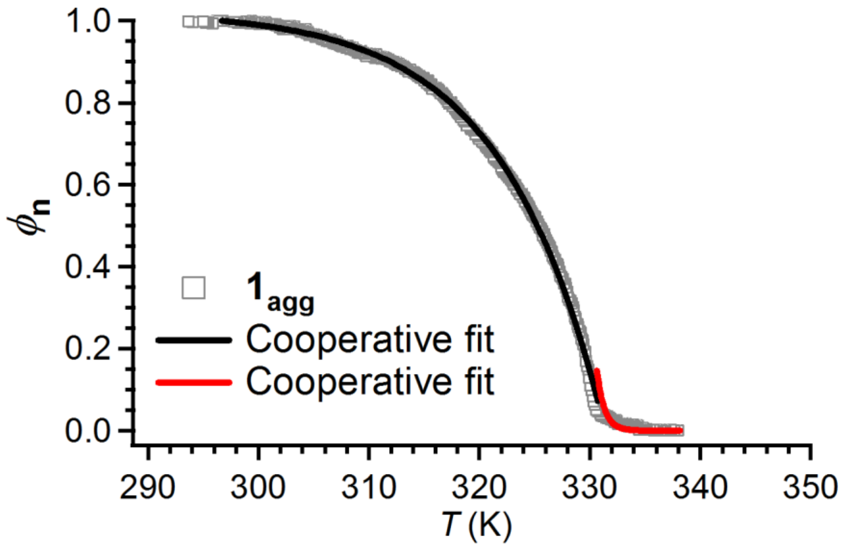

Supplementary Figure 4. Cooling curve obtained by UV study of 1. Normalized UV intensity (ϕ_n) at 365 nm as a function of the temperature, obtained by monitoring the UV intensity of MCH solutions of **1** ([**1**] = 1.0 × 10^{-4} M) upon cooling hot monomeric solutions to 293 K with a cooling rate of 1 K·min^{-1}. The curve could be fitted using cooperative model.

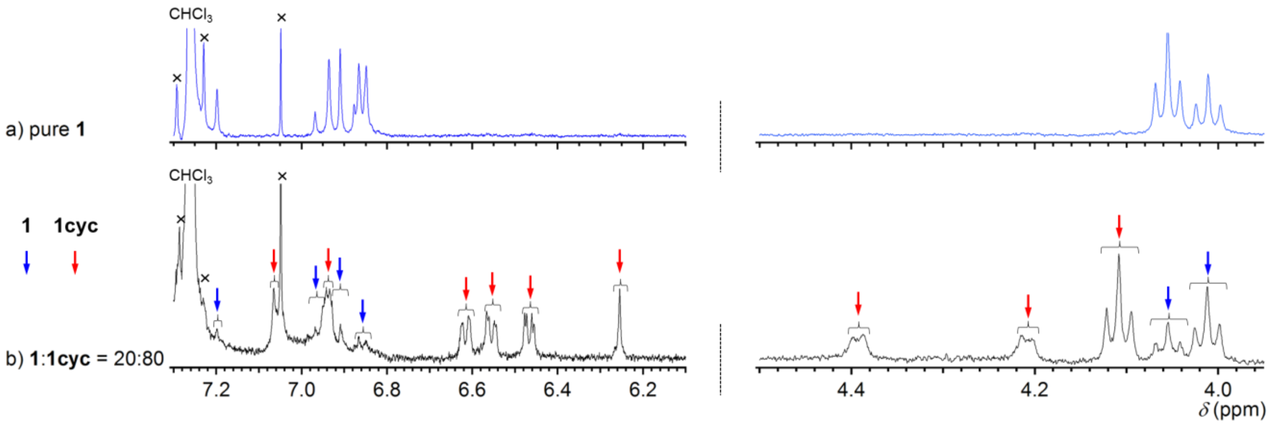

Supplementary Figure 5. ^{1}H NMR spectra of 1 with 1cyc. (**a**,**b**) ^{1}H NMR spectra of (**a**) pure **1** and (**b**) **1** with 80% **1cyc**, obtained by UV-irradiation of **1**$_{\mathbf{agg}}$ (in MCH at 293 K), in $CDCl_3$. Blue and red arrows show signals corresponding to **1** and **1cyc**, respectively.

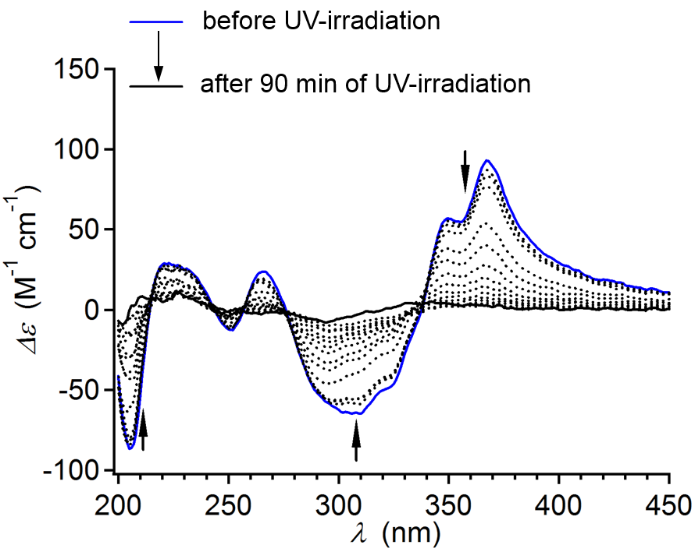

Supplementary Figure 6. Change of the CD spectra of 1 upon exposure to UV-irradiation. Change of the CD spectra of **1** ([**1**] = 1.0 × 10^{-4} M) in MCH upon exposure to UV-irradiation for 90 min at 293 K.

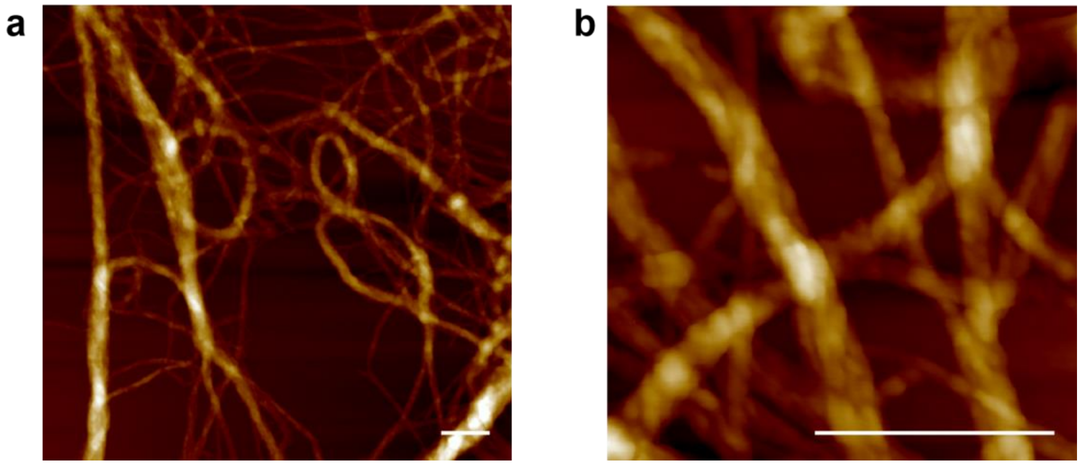

Supplementary Figure 7. AFM images of (1:1cyc$_{f=0.8}$)$_{agg}$. (**a**,**b**) AFM height images of (**1**:**1cyc**$_{f=0.8}$)$_{agg}$, spin-coated from MCH solution (total concentration = 1.0 × 10^{-4} M) at 293 K onto HOPG. Scale bars, 500 nm.

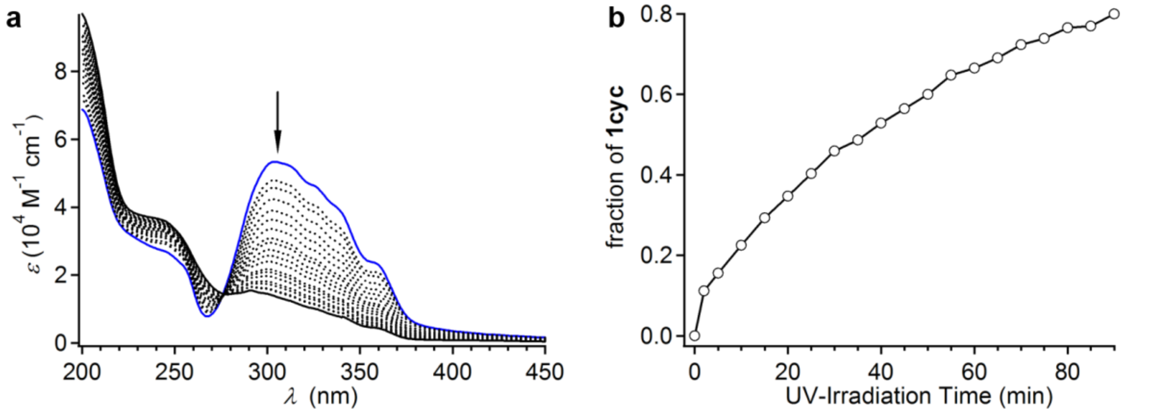

Supplementary Figure 8. UV spectra and plot of fraction of 1cyc versus UV-irradiation time upon UV-irradiation of 1_{agg}. (**a**) UV spectra and (**b**) plot of fraction of **1cyc** versus UV-irradiation time upon UV-irradiation of $\mathbf{1_{agg}}$ in MCH at 293 K at total concentration = 1.0 × 10^{-4} M.

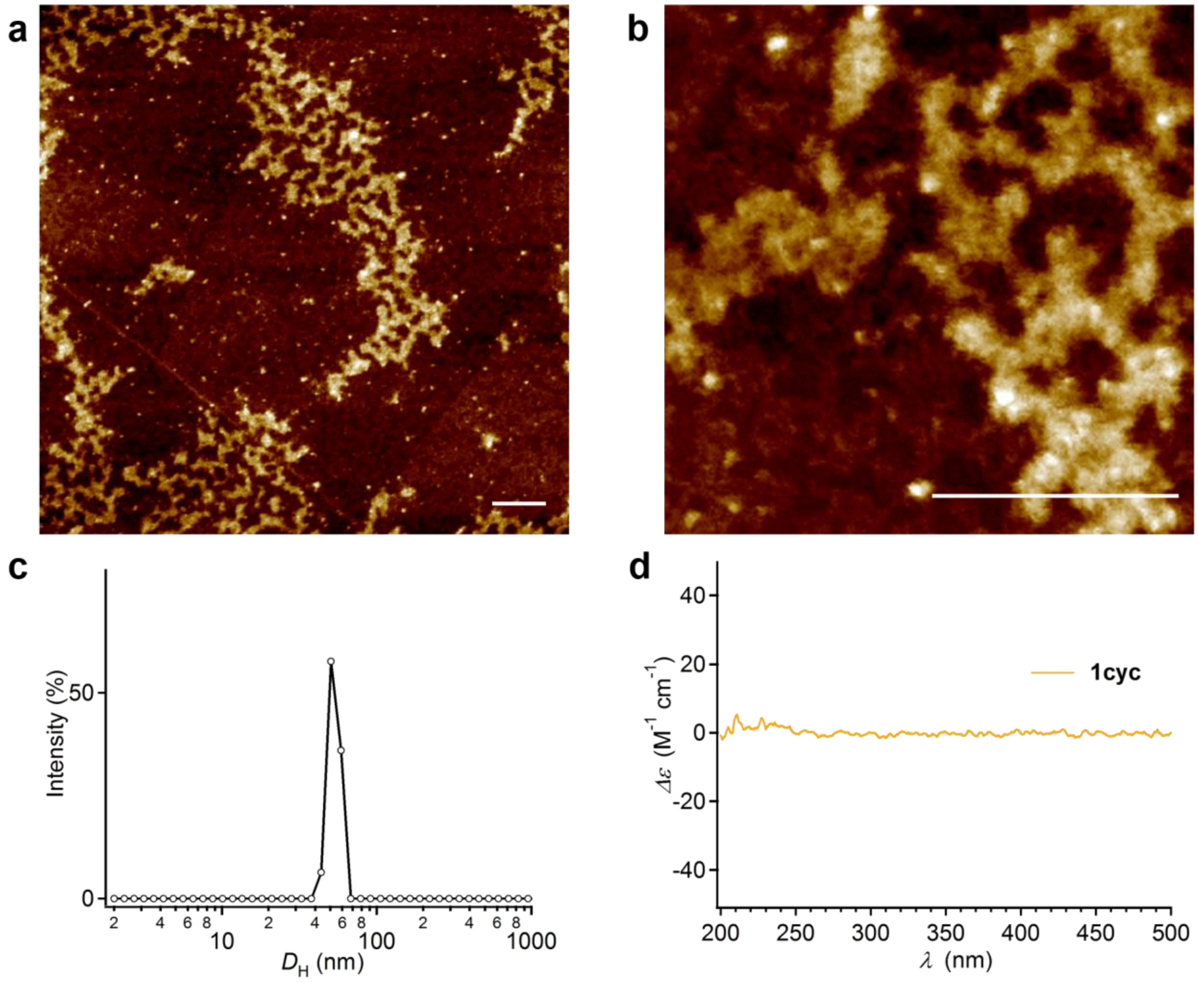

Supplementary Figure 9. AFM, DLS and CD studies of 1cyc. (**a**,**b**) AFM height images of **1cyc**, spin-coated from MCH solution ([**1cyc**] = 1.0 × 10^{-4} M) at 293 K onto HOPG. Scale bars, 100 nm. (**c**) DLS and (**d**) CD results of **1cyc** in MCH ([**1cyc**] = 1.0 × 10^{-4} M) at 293 K.

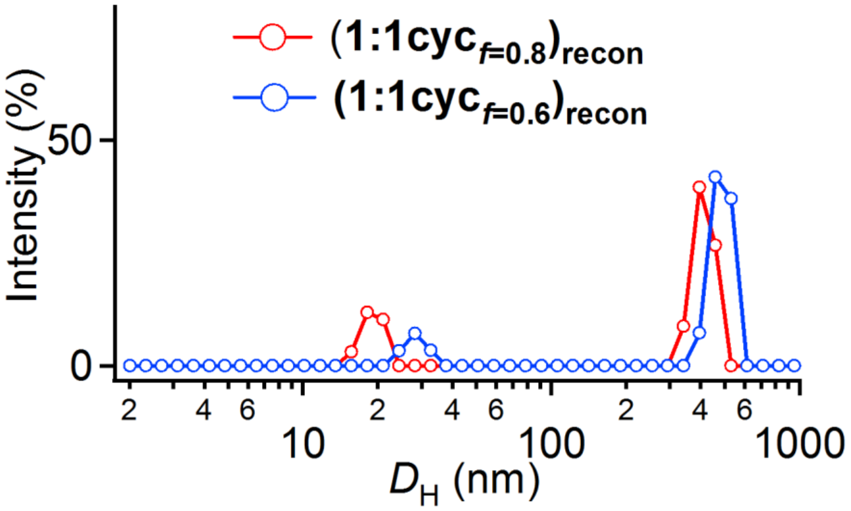

Supplementary Figure 10. DLS results for (1:1cyc$_{f=0.6,0.8}$)$_{recon}$. DLS results of **(1:1cyc$_{f=0.6,0.8}$)$_{recon}$** in MCH at 293 K.

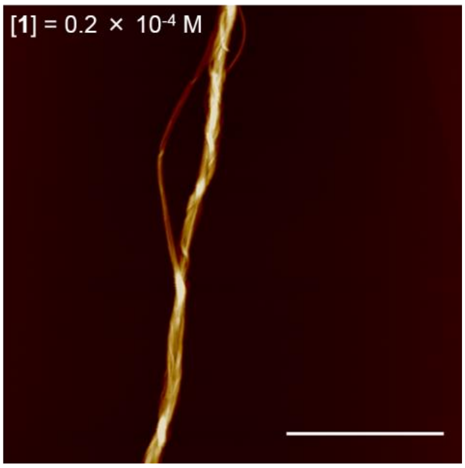

Supplementary Figure 11. AFM image of 1$_{agg}$. AFM height images of **1**$_{agg}$, spin-coated from MCH solutions at two different concentrations ([**1**] = 2.0 × 10^{-5} M) at 293 K onto HOPG. Scale bar, 1 µm.

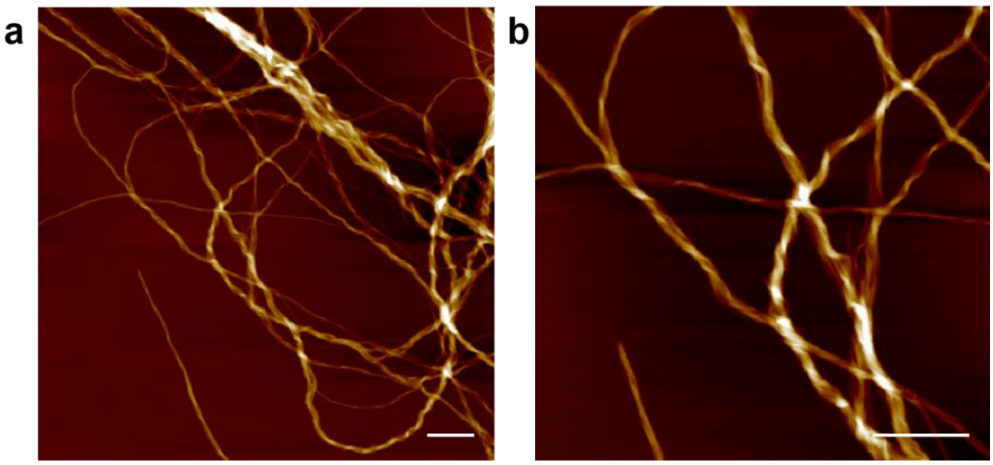

Supplementary Figure 12. AFM image of (1:1cyc$_{f=0.25}$)$_{recon}$. (**a**,**b**) AFM height images of **(1:1cyc$_{f=0.25}$)$_{recon}$**, spin-coated from a MCH solution (total concentration = 1.0×10^{-4} M) at 293 K onto HOPG. Scale bars, 500 nm.

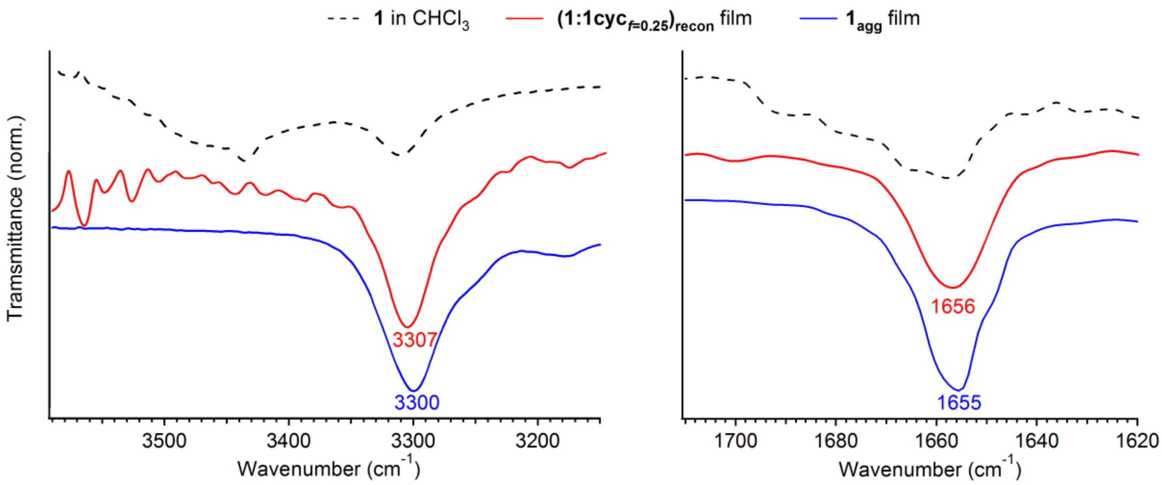

Supplementary Figure 13. IR spectra. IR spectra of **1** ([**1**] = 10 mM) in $CHCl_3$ (dashed curve), **(1:1cyc$_{f=0.25}$)$_{recon}$** (red curve) and **1$_{agg}$** (blue curve).

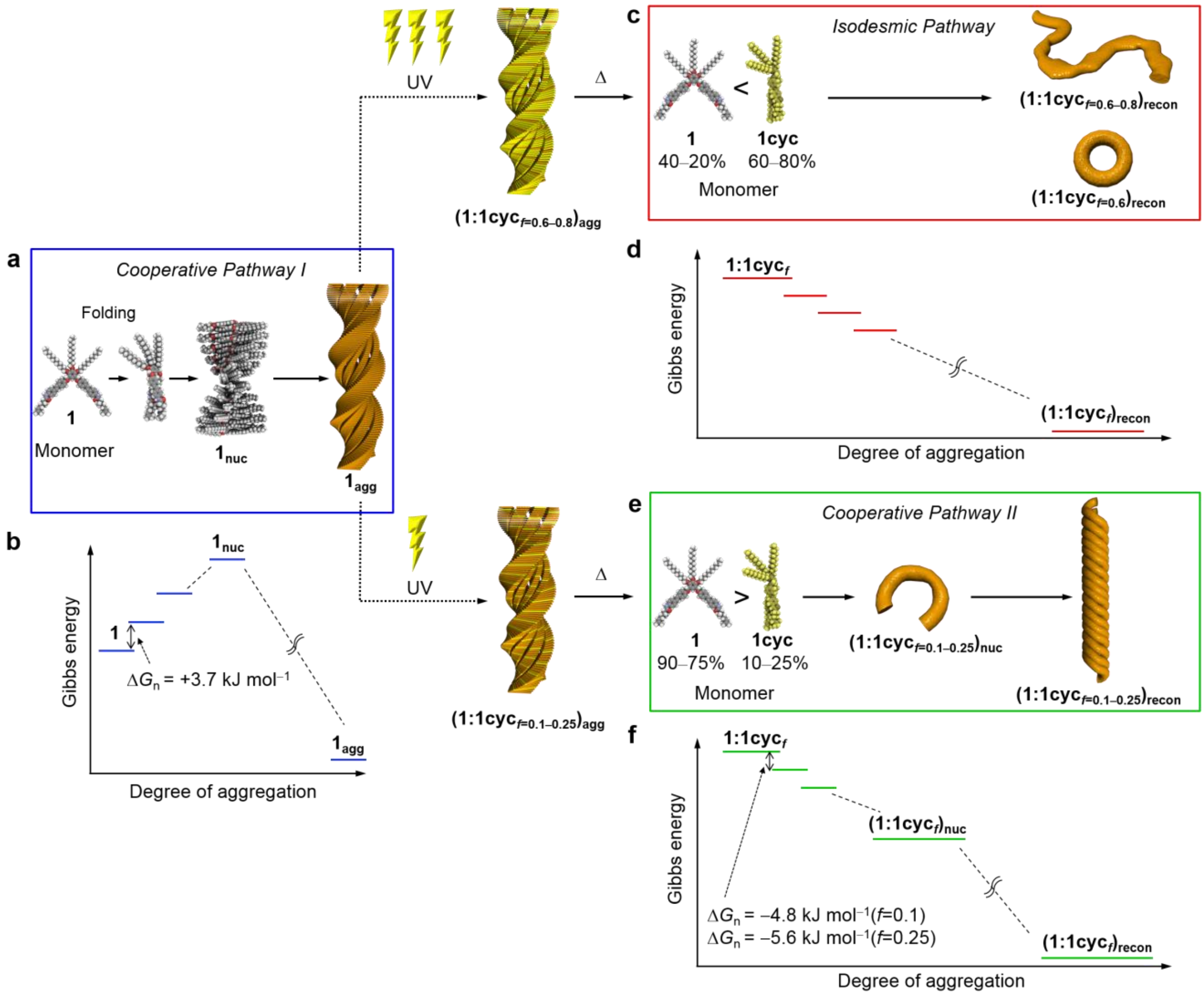

Supplementary Figure 14. Photoreaction-guided (co)assembly pathways of 1. (**a**,**c**,**e**) Schematic representation of three (co)assembly pathways of **1** and **1cyc**. (**b**,**d**,**f**) Energy landscape for the three assembly pathways of **1** and **1cyc**. (**a**,**b**): Cooperative assembly pathway for $\mathbf{1}_{\mathbf{agg}}$; (**c**,**d**): isodesmic co-assembly pathway for $\mathbf{(1{:}1cyc}_{f=0.6\text{–}0.8}\mathbf{)}_{\mathbf{recon}}$; (**e**,**f**): downhill cooperative co-assembly pathway for $\mathbf{(1{:}1cyc}_{f=0.1\text{–}0.25}\mathbf{)}_{\mathbf{recon}}$.

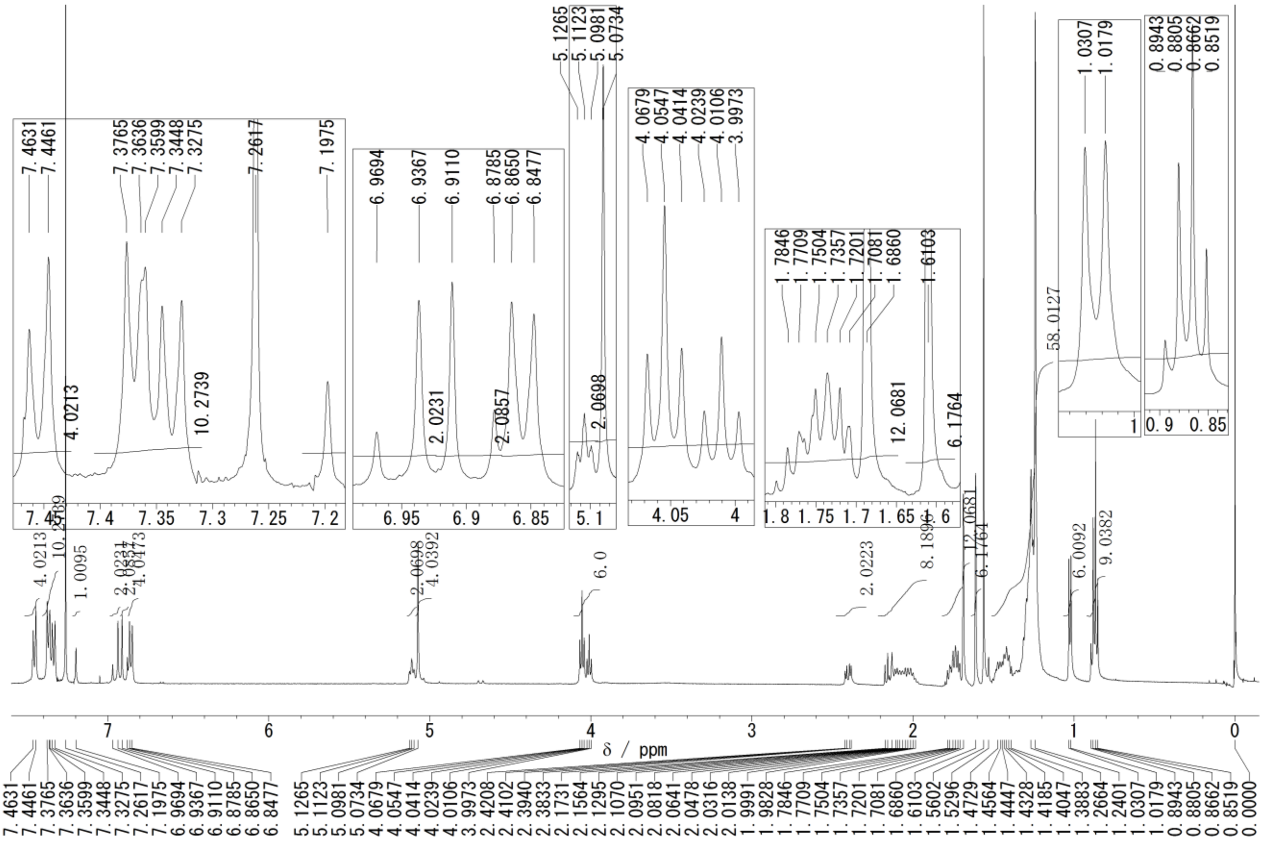

Supplementary Figure 15. ^{1}H NMR spectrum of 1. ^{1}H NMR spectrum of **1** in $CDCl_3$ at 293 K.

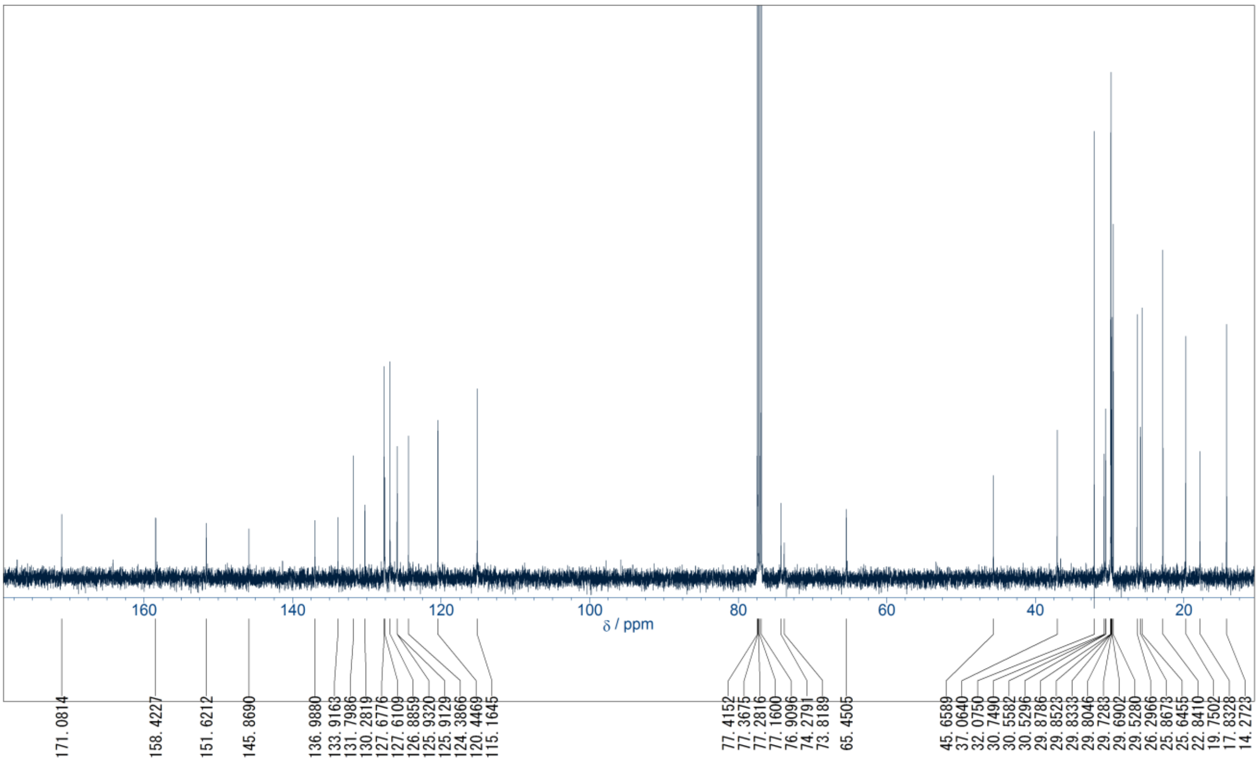

Supplementary Figure 16. ^{13}C NMR spectrum of 1. ^{13}C NMR spectrum of **1** in $CDCl_3$ at 293 K.

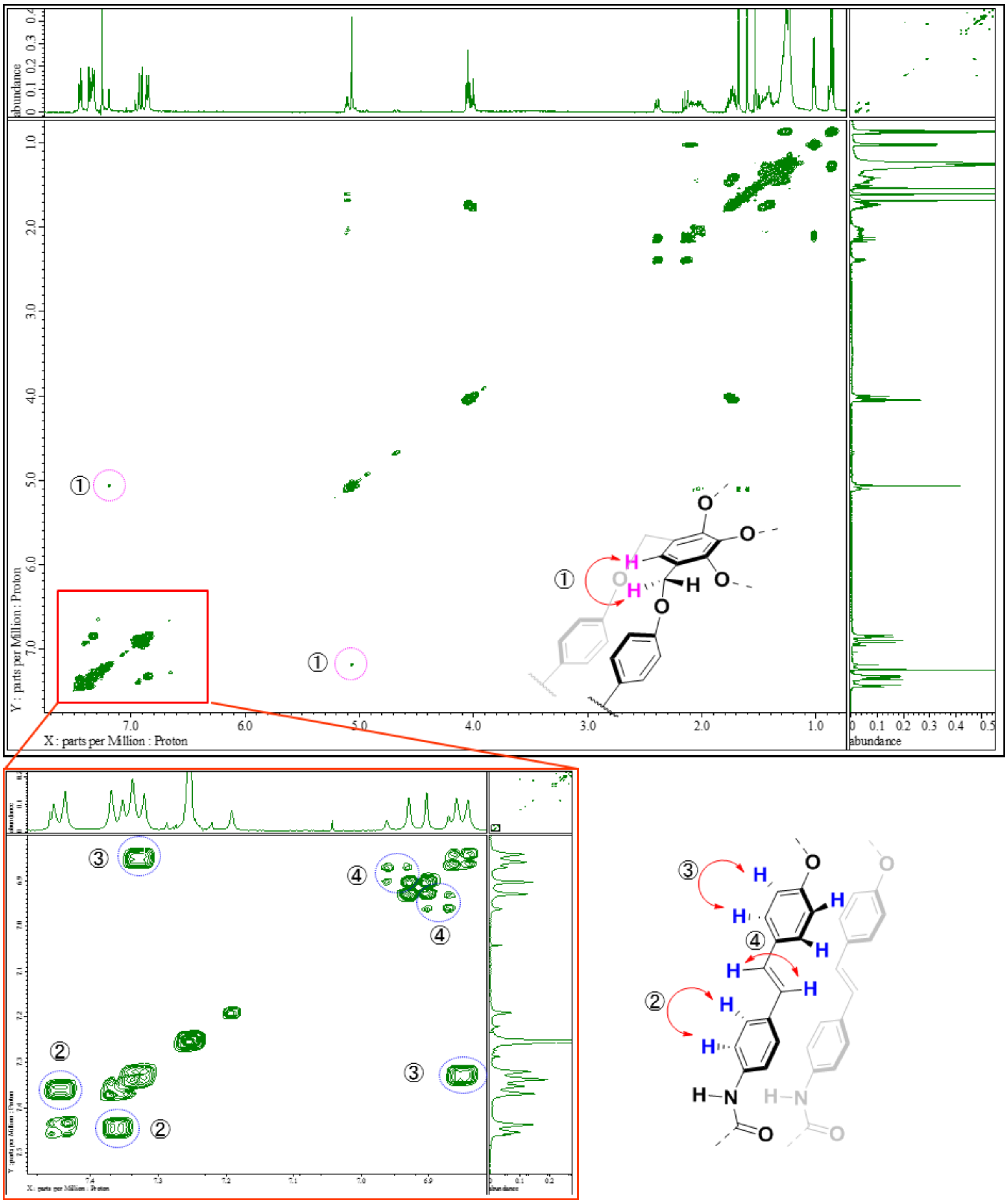

Supplementary Figure 17. 2D COSY NMR spectrum of 1. 2D COSY NMR spectrum of **1** in $CDCl_3$ at 293 K.

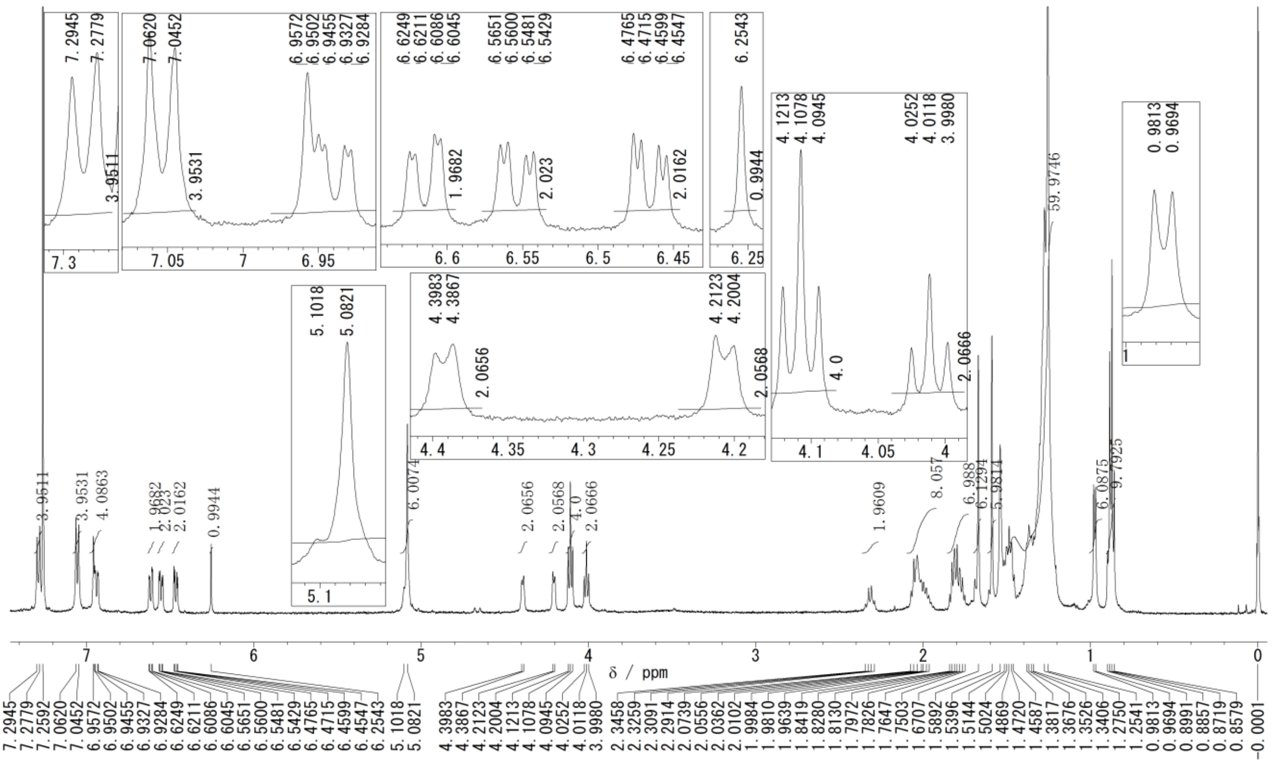

Supplementary Figure 18. ^{1}H NMR spectrum of 1cyc. ^{1}H NMR spectrum of **1cyc** in $CDCl_3$ at 293 K.

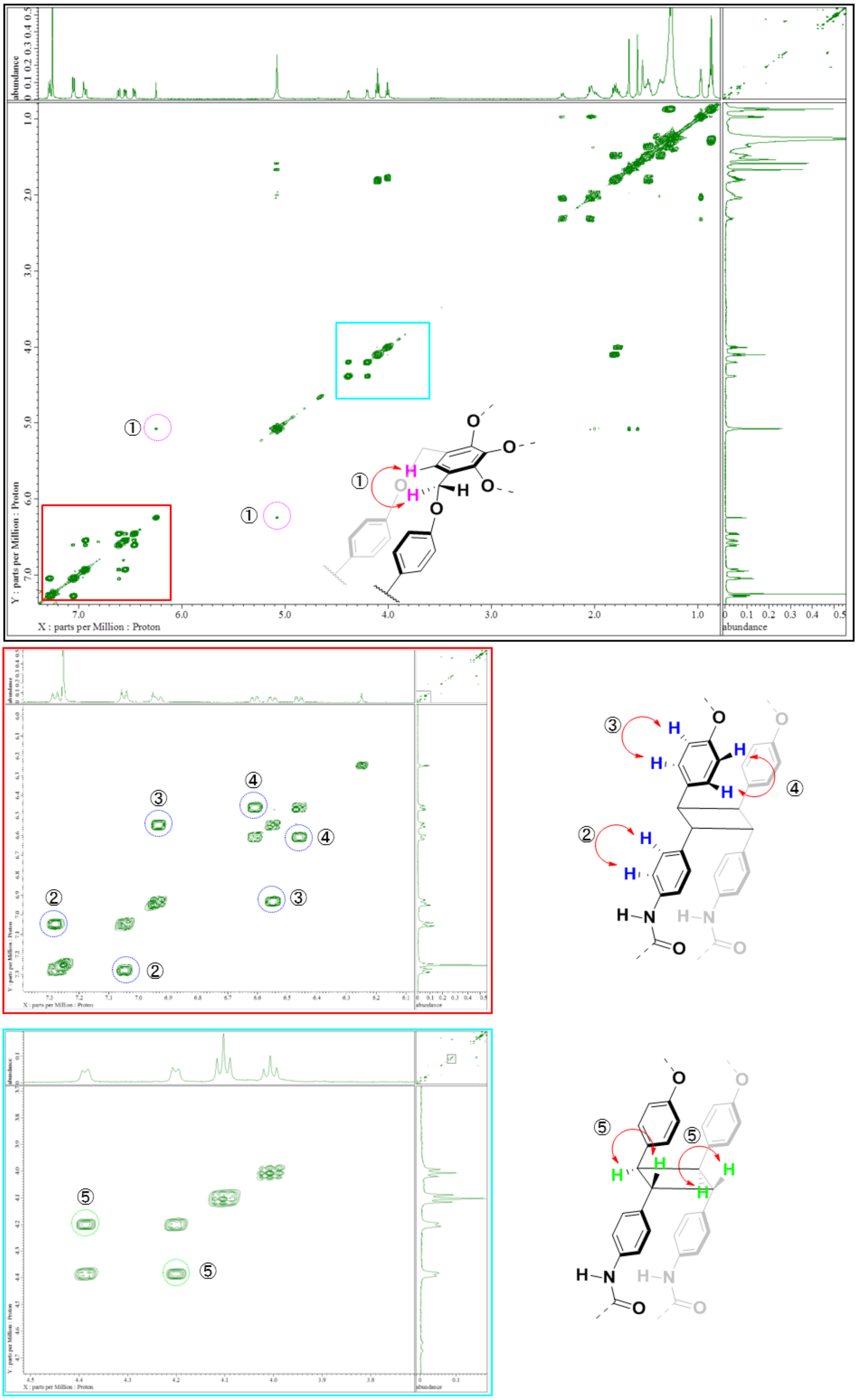

Supplementary Figure 19. 2D COSY NMR spectrum of 1cyc. 2D COSY NMR spectrum of **1cyc** in $CDCl_3$ at 293 K.

Supplementary Table 1. Thermodynamic parameters for the self-assembly of 1. Thermodynamic parameters for the cooperative self-assembly of **1** in MCH, estimated from temperature-dependent CD spectra measured at varying concentrations.

[**1**] (M)	T_e (K)	ΔH_e (kJ mol^{-1})	K_a
1.3×10^{-4}	330.0	-84.6 ± 0.2	1.1×10^{-5}
1.0×10^{-4}	327.7	-78.1 ± 0.4	2.6×10^{-5}
0.8×10^{-4}	325.1	-72.4 ± 0.4	2.2×10^{-5}
0.4×10^{-4}	318.0	-86.7 ± 2.2	4.4×10^{-5}

Supplementary Table 2. Thermodynamic parameters for the isodesmic co-assemblies of 1 and 1cyc. Thermodynamic parameters for the isodesmic assembly process of **1** in the presence of major amounts of **1cyc** in MCH, determined by fitting the temperature-dependent CD data.

	fraction of **1cyc**	T_m (K)	ΔH_{iso} (kJ mol^{-1})	K_{iso} (M^{-1})^a
**(1:1cyc$_{f=0.6}$)$_{recon}$**	0.6	317.2	-133.7 ± 0.6	8.6×10^4
**(1:1cyc$_{f=0.8}$)$_{recon}$**	0.8	312.7	-136.8 ± 1.4	8.3×10^4

^{a} values determined at 298 K.

Supplementary Table 3. Thermodynamic parameters for the cooperative (co)assemblies of 1 and 1cyc. Thermodynamic parameters for the cooperative assembly process of **1** in the presence of minor amounts of **1cyc** in MCH, determined by fitting the temperature-dependent CD data.

	fraction of **1cyc**	T_e (K)	ΔH_e (kJ mol^{-1})	K_a
**1$_{agg}$**	0	327.7	-78.1 ± 0.4	2.6×10^{-5}
**(1:1cyc$_{f=0.1}$)$_{recon}$**	0.1	327.9	-187.8 ± 0.8	5.9×10^{-4}
**(1:1cyc$_{f=0.25}$)$_{recon}$**	0.25	328.4	-128.9 ± 0.6	7.9×10^{-4}

Supplementary Methods

General: All commercially available reagents and solvents were of reagent grade and used without purification. The solvents for the preparation of the assemblies were all spectral grade and used without purification. ^{1}H and ^{13}C NMR, and 2D-COSY NMR spectra were recorded on JEOL JNM-ECA500 NMR spectrometer and chemical shifts are reported in ppm (δ) with the signal of TMS as the internal standard. ESI-MS spectra were measured on an Exactive (Thermo Scientific). UV/vis and circular dichroism (CD) spectra were recorded on a JASCO V660 spectrophotometer and JASCO J840 spectropolarimeter, respectively. Both the spectrometers are equipped with Peltier device temperature-control unit. Dynamic light scattering (DLS) measurements were performed on a Zetasizer Nano S (Malvern Instruments) device using non-invasive back-scatter technology (NIBS) under 4.0 mW He-Ne laser (633 nm). The scattering angle was set at 173°. FT/IR spectra were recorded on JASCO FT/IR-4100 spectrometer with an attenuated total reflectance (ATR) apparatus. TEM observation was performed on JEM-2100F (JEOL) at acceleration voltage at 120 kV. Samples were prepared by spin-coating assembly solutions onto carbon-coated STEM Cu grid (SHR-C075) and dried under vacuum for 24 h.

Synthesis: Stilbene dyad **1** was synthesized according to:

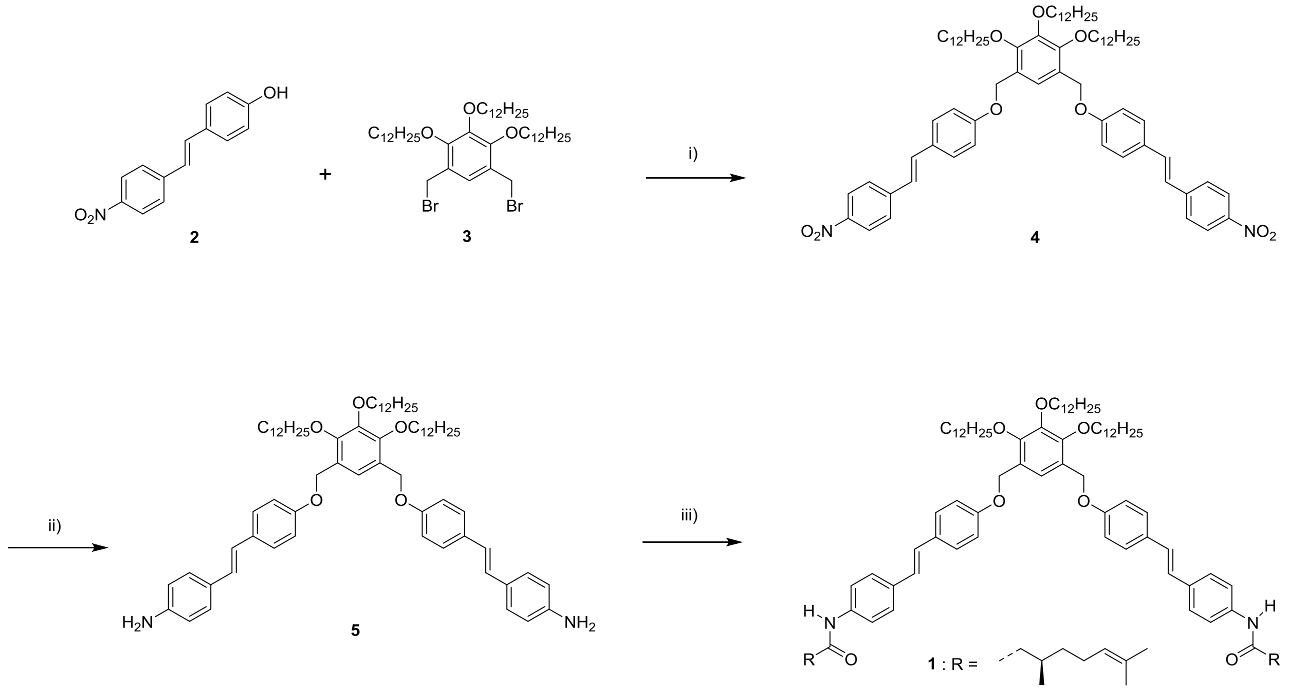

i) K_2CO_3, DMF, 70 °C; ii) Na_2S, 1,4-dioxane, H_2O, 100 °C; iii) (*R*)-(+)-citronellic acid, DCC, DMAP, CH_2Cl_2, r.t..

Synthesis of 4: Compound **2** (270 mg, 1.12 mmol) was added to a suspension of K_2CO_3 (342 mg, 2.5 mmol) in dry DMF (10 mL) at 70 °C. To this solution, 1,5-bis(bromomethyl)-2,3,4-tris(dodecyloxy)benzene[1] (**3**, 457 mg, 0.56 mmol) dissolved in dry DMF (5 mL) was added dropwise and the mixture was stirred for 18 h at 70 °C under N_2 atmosphere. The reaction mixture was poured into ice-water and the resulting precipitates were purified by column chromatography over silica gel (eluent: $CHCl_3$) to give **4** as a yellow solid (534 mg, 84% yield). ^{1}H NMR (500 MHz, $CDCl_3$, 298 K): δ 8.19 (4H, d, J = 8.8 Hz, PhH_a), 7.58 (4H, d, J = 8.8 Hz, PhH_b), 7.48 (4H, d, J = 8.8 Hz, PhH_c), 7.28 (1H, s, PhH_d), 7.22 (2H, d, J = 16.3 Hz, PhCH_e=CHPh), 7.00 (2H, d, J = 16.3 Hz, PhCH=CH_fPh), 6.99 (4H, d, J = 8.8 Hz, PhH_g), 5.06 (4H, s, PhCH_h), 4.08 (4H, t, J = 6.6 Hz,

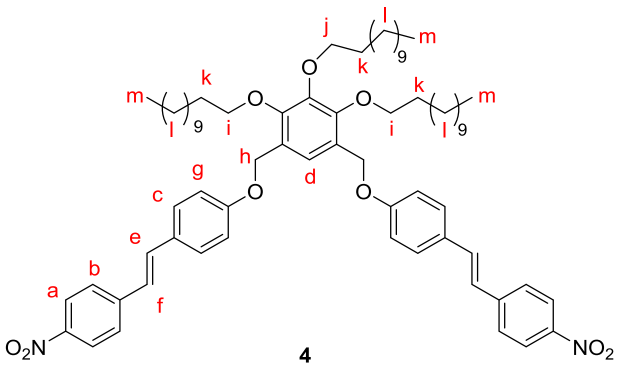

CH_i), 4.02 (2H, t, J = 6.6 Hz, CH_j), 1.79–1.69 (6H, m, CH_k), 1.48–1.22 (54H, m, CH_l), 0.89–0.84 (9H, m, CH_m). ^{13}C NMR (125 MHz, $CDCl_3$, 298 K): δ 159.70, 152.18, 146.52, 145.92, 144.41, 133.05, 129.18, 128.56, 126.61, 125.65, 124.28, 124.19, 115.23, 74.43, 73.84, 65.45, 32.06, 30.53, 30.51, 29.88, 29.84, 29.82, 29.79, 29.70, 29.67, 29.53, 26.31, 26.29, 22.83, 14.24. HRMS (ESI): *m/z* calcd for $C_{72}H_{100}O_9N_2Cl$ 1171.7123 $[M+Cl]^-$, found 1171.7159.

Synthesis of 5: Compound **4** (150 mg, 0.132 mmol) was dissolved in 1,4-dioxane (5 mL) at 65 °C under N_2 atmosphere. To this solution, Na_2S (500 mg, 2 mmol) dissolved in a mixture of water (1.0 mL) and 1,4-dioxane (5 mL) was added drop-wise, and the mixture was stirred for 6.5 h at 100 °C. After the reaction mixture was cooled to r.t., 1,4-dioxane was removed by evaporation. The resulting solid was dissolved in $CHCl_3$ and washed with water. The organic layers were dried over Na_2SO_4 and evaporated to dryness. The resulting solid was reprecipitated from $CHCl_3$-MeOH mixture to give **5** as an orange solid (142 mg, *ca.* 100% yield). ^{1}H NMR (500 MHz, $CDCl_3$, 298 K): δ 7.38 (4H, d, J = 8.8 Hz, PhH_a), 7.30 (4H, d, J = 8.6 Hz, PhH_b), 7.28 (1H, s, PhH_c), 6.93 (4H, d, J = 8.8 Hz, PhH_d), 6.88–6.87 (4H, m, PhCH_e=CH_ePh), 6.66 (4H, d, J = 8.6 Hz, PhH_f), 5.03 (4H, s, PhCH_g), 4.06 (4H, t, J = 6.6 Hz, CH_h), 4.02 (2H, t, J = 6.6 Hz, CH_i), 1.77–1.70 (6H, m, CH_j), 1.40–1.24 (54H, m, CH_k), 0.89–0.86 (9H, m, CH_l). HRMS (ESI): *m/z* calcd for $C_{72}H_{105}O_5N_2$ 1077.8018 $[M+H]^+$, found 1077.8018. ^{13}C NMR spectrum of this compound could not be measured due to its very low solubility.

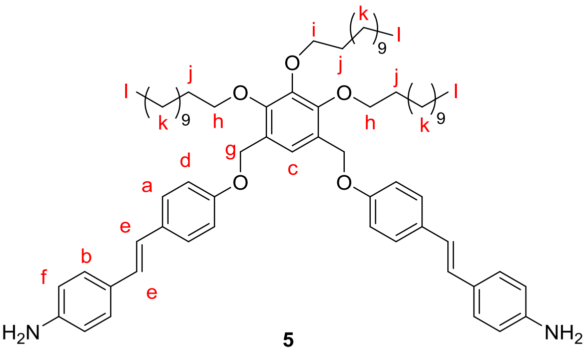

Synthesis of 1: Compound **5** (84 mg, 0.077 mmol), (*R*)-(+)-citronellic acid (26 mg, 0.15 mmol) and *N*,*N*-dimethyl-4-aminopyridine (DMAP, 2 mg, 0.018 mmol) were dissolved in dry CH_2Cl_2 (3.0 mL). A solution of *N*,*N*′-dicyclohexylcarbodiimide (DCC, 31 mg, 0.15 mmol) in dry CH_2Cl_2 (2 mL) was added drop-wise at 0 °C, and the mixture was stirred for 17 h at room temperature under N_2 atmosphere. After the resulting white precipitates were filtered off, the filtrate was diluted with CH_2Cl_2 and washed with brine. The organic layer was dried over Na_2SO_4 and evaporated to dryness. The resulting solid was reprecipitated from $CHCl_3$–MeOH mixture and further purified by column chromatography over silica gel ($CHCl_3$:ethyl acetate = 20:1) to give **1** as a yellow solid (33 mg, 31% yield). ^{1}H NMR (500 MHz, $CDCl_3$, 298 K): δ 7.45 (4H, d, J = 8.5 Hz, PhH_a), 7.36 (4H, d, J = 8.5 Hz, PhH_b), 7.36 (2H, s, amide-H_c), 7.33 (4H, d, J = 8.8 Hz, PhH_d), 7.20 (1H, s, PhH_e), 6.95 (2H, d, J = 16.3 Hz, PhCH=CH_fPh), 6.89 (2H, d, J = 16.3 Hz, PhCH_g=CHPh), 6.85 (4H, d, J = 8.8 Hz, PhH_h), 5.11 (2H, t, J = 7.2 Hz, CH_i=C(CH_3)$_2$), 5.07 (4H, s, PhCH_j), 4.05 (4H, t, J = 6.6 Hz, CH_k), 4.01 (2H, t, J = 6.6 Hz, CH_l), 2.42–1.98 (10H, m, C$H_{m–o}$), 1.78–1.71 (6H, m, CH_p), 1.69 (6H, s, CH_q), 1.61 (6H, s, CH_r), 1.47–1.24 (58H, m, C$H_{s,t}$), 1.02 (6H, d, J = 6.4 Hz, CH_u), 0.90–0.85 (9H, m, CH_v). ^{13}C NMR (125 MHz, $CDCl_3$, 298 K): δ 171.08, 158.42, 151.62, 145.87, 136.99, 133.91, 131.80, 130.28, 127.68, 127.61, 126.88, 125.93, 125.91, 124.38, 120.44, 115.16, 74.28, 73.82, 65.45, 45.66, 37.06, 32.07, 30.75, 30.56, 30.53, 29.88, 29.85, 29.83, 29.80, 29.73, 29.69, 29.53, 26.29, 25.86, 25.64, 22.84, 19.75, 17.83, 14.27. HRMS (ESI): *m*/*z* calcd for $C_{92}H_{137}O_7N_2$ 1382.0420 [M+H]$^+$, found 1382.0416.

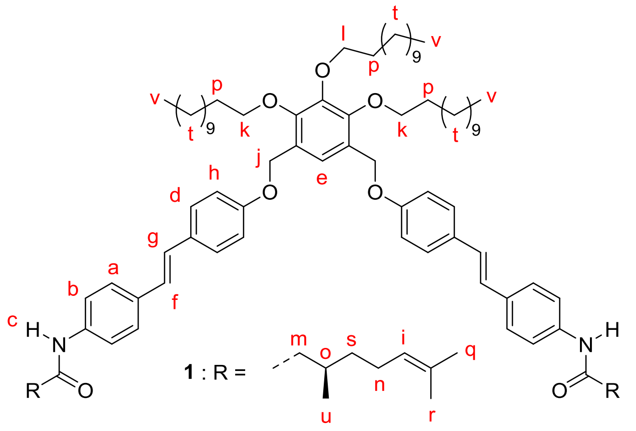

Characterization of photoproduct 1cyc: ^{1}H NMR (500 MHz, $CDCl_3$, 298 K): δ 7.28 (4H, d, J = 8.4 Hz, PhH_a), 7.05 (4H, d, J = 8.4 Hz, PhH_b), 6.96 (2H, s, amide-H_c), 6.94 (2H, dd, J = 8.6, 2.2 Hz, PhH_d), 6.61 (2H, dd, J = 8.4, 2.1 Hz, PhH_e), 6.55 (2H, dd, J = 8.5, 2.4 Hz, PhH_f), 6.46 (2H, dd, J = 8.4, 2.6 Hz, PhH_g), 6.25 (1H, s, PhH_h), 5.10 (2H, br, CH_i=C(CH_3)$_2$), 5.08 (4H, s, PhCH_j), 4.39 (2H, d, J = 5.9 Hz, cyclobutane-H_k), 4.20 (2H, d, J = 5.9 Hz, cyclobutane-H_l), 4.05 (4H, t, J = 6.6 Hz, CH_m), 4.01 (2H, t, J = 6.6 Hz, CH_n), 2.35–1.96 (10H, m, C$H_{o\text{–}q}$), 1.84–1.75 (6H, m, CH_r), 1.67 (6H, s, CH_s), 1.59 (6H, s, CH_t), 1.51–1.25 (58H, m, C$H_{u,v}$), 0.97 (6H, d, J = 5.9 Hz, CH_w), 0.90–0.86 (9H, m, CH_x). HRMS (ESI): m/z calcd for $C_{92}H_{137}O_7N_2$ 1382.0420 [M+H]$^+$, found 1382.0449.

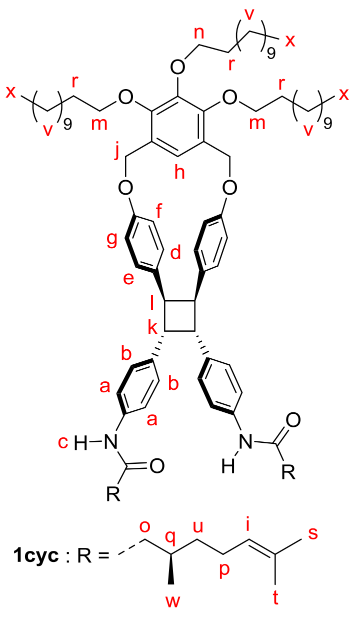

Assembly models:

Calculation of ϕ_n: Normalized CD intensities (ϕ_n) were calculated from CD intensities of temperature-dependent CD spectra at appropriate wavelength based on equation (1), in which CD_{agg} and CD_{mon} are the CD intensities of fully aggregated (the lowest temperature) and pure monomeric species (the highest temperature), respectively, and $CD(T)$ is the CD intensity at a given temperature.

$$\phi_n(T) = 1 - \frac{CD_{agg} - CD(T)}{CD_{agg} - CD_{mon}} \qquad (1)$$

Isodesmic (equal-*K*) model: If a plot of ϕ_n *versus* T shows sigmoidal relation, the aggregation can be characterized as isodesmic process[2,3], and the plot can be fitted with equation (2):

$$\phi_n(T) = \frac{1}{1 + \exp[-0.908\Delta H_{iso} \frac{T - T_m}{RT_m{}^2}]} \qquad (2)$$

where ΔH_{iso} is the enthalpy release during isodesmic aggregation and T_m is the melting temperature for $\phi_n = 0.5$.

Degree of polymerization (DP_n) for isodesmic model can be described by equation (3):

$$DP_n(T) = \frac{1}{\sqrt{1 - \phi_n(T)}} = \frac{1}{2} - \frac{1}{2}\sqrt{4K_{iso}(T)C_T + 1} \qquad (3)$$

where $K_{iso}(T)$ is the temperature-dependent equilibrium constant and C_T is the total concentration. Thus, $K_{iso}(T)$ can be obtained bywith equation (4).

$$K_{iso}(T) = \frac{(\frac{2}{\sqrt{1 - \phi_n(T)}} - 1)^2 - 1}{4C_T} \qquad (4)$$

Cooperative (nucleation–elongation) model: Cooperative assembly processes were analyzed by the nucleation–elongation model developed by van der Schoot, Meijer, Schenning and coworkers.[4,5] If a curve obtained by plotting ϕ_n *versus* T in temperature-dependent CD analysis is non-sigmoidal, the curve can be fitted with a cooperative model, which involves two different regimes expressed by following two equations (5) and (6). The two different regimes are defined as the nucleation and the elongation processes, respectively, which are divided by onset temperature for the elongation (T_e).

In the elongation regime ($T < T_e$), ϕ_n is given by the following equation (5):

$$\phi_n(T) = \phi_{SAT}[1 - \exp\left[\frac{-\Delta H_e(T - T_e)}{RT_e^2}\right]] \qquad (5)$$

where ΔH_e is the enthalpy release in the elongation regime, ϕ_{SAT} is a parameter introduced to ensure that ϕ_n/ϕ_{SAT} does not exceed unity, R is the ideal gas constant. Therefore, the nonlinear least-squares analysis directly affords ΔH_e, T_e and ϕ_{SAT}.

In the nucleation regime ($T > T_e$), ϕ_n is given by the following equation (6):

$$\phi_n(T) = \phi_{SAT}[K_a^{\frac{1}{3}} \exp[\left(\frac{2}{3K_a^{\frac{1}{3}}} - 1\right)\Delta H_e\left[\frac{(T - T_e)}{RT_e^2}\right]]] \qquad (6)$$

where K_a is the dimensionless activation equilibrium constant at T_e, and can be obtained by nonlinear least-squares analysis. The nucleation step is governed by K_a, a parameter reflecting degree of cooperativity. If $K_a << 1$, the aggregation process can be characterized by a model with high cooperativity. In this case, the critical concentration of monomer is approximately total concentration (C_T) at $\geq T_e$, thereby the elongation constant denoted as K_e is equated to the inverse of C_T.[4] Because

K_e is governed by free energy change of the elongation process (ΔG_e), R and T, $K_e(T_e)$ is given by the following equation (7):

$$K_e(T_e) = \exp\left[\frac{-\Delta G_e}{RT_e}\right] = \frac{1}{C_T} \tag{7}$$

Assuming that K_a is equal to K_n/K_e in the nucleation regime at T_e,[6] the free energy changes of nucleation (ΔG_n) at $>T_e$ is given by the following equation (8):

$$\begin{aligned}\Delta G_n(T_e) &= -RT_e \ln(K_n) = -RT_e \ln(K_a K_e)\\ &= -RT_e \ln\left(\frac{K_a}{C_T}\right)\end{aligned} \tag{8}$$

Supplementary References

1. Yao, S., Beginn, U., Gress, T., Lysetska, M. & Würthner, F. Supramolecular polymerization and gel formation of bis(merocyanine) dyes driven by dipolar aggregation. *J. Am. Chem. Soc.* **126,** 8336–8348 (2004).
2. Martin, R. B. Comparisons of indefinite self-association models. *Chem. Rev.* **96,** 3043–3064 (1996).
3. Smulders, M. M. J. *et al.* How to distinguish isodesmic from cooperative supramolecular polymerisation. *Chem. Eur. J.* **16,** 362–367 (2010).
4. Van der Schoot, P. Chapter 3 Theory of supramolecular polymerization in *SUPRAMOLECULAR POLYMERS,* A. Ciferri, Ed. (CRC Press, Baton Rouge, LA, 2005).
5. Jonkheijm, P., Schoot, P. van der, Schenning, A. P. H. J. & Meijer, E. W. Probing the solvent-assisted nucleation pathway in chemical self-assembly. *Science* **313,** 80–83 (2006).
6. García, F. & Sánchez, L. Structural rules for the chiral supramolecular organization of OPE-based discotics: induction of helicity and amplification of chirality. *J. Am. Chem. Soc.* **134,** 734–742 (2012).
